# The role of seagrass vegetation and local environmental conditions in shaping benthic bacterial and macroinvertebrate communities in a tropical coastal lagoon

**DOI:** 10.1038/s41598-020-70318-1

**Published:** 2020-08-11

**Authors:** Z. Alsaffar, J. K. Pearman, J. Cúrdia, J. Ellis, M. Ll. Calleja, P. Ruiz-Compean, F. Roth, R. Villalobos, B. H. Jones, X. A. G. Morán, S. Carvalho

**Affiliations:** 1 Biological and Environmental Sciences and Engineering (BESE), Red Sea Research Center (RSRC), King Abdullah University of Science and Technology (KAUST), Thuwal, 23955-6900 Saudi Arabia; 2 King Saud University, Riyadh, Saudi Arabia; 3 Coastal and Freshwater Group, Cawthron Institute, Nelson, New Zealand; 4 School of Science, University of Waikato, Tauranga, New Zealand; 5 Department of Climate Geochemistry, Max Planck Institute for Chemistry (MPIC), Mainz, Germany; 6 Baltic Sea Centre, Stockholm University, Stockholm, Sweden; 7 Faculty of Biological and Environmental Sciences, Tvärminne Zoological Station, University of Helsinki, Helsinki, Finland

**Keywords:** Ecology, Microbiology, Ecology, Environmental sciences

## Abstract

We investigated the influence of seagrass canopies on the benthic biodiversity of bacteria and macroinvertebrates in a Red Sea tropical lagoon. Changes in abundance, number of taxa and assemblage structure were analyzed in response to seagrass densities (low, SLD; high, SHD; seagrasses with algae, SA), and compared with unvegetated sediments. Biological and environmental variables were examined in these four habitats (hereafter called treatments), both in the underlaying sediments and overlaying waters, at three randomly picked locations in March 2017. Differences between treatments were more apparent in the benthic habitat than in the overlaying waters. The presence of vegetation (more than its cover) and changes in sedimentary features (grain size and metals) at local scales influenced the observed biological patterns, particularly for macroinvertebrates. Of note, the highest percentage of exclusive macroinvertebrate taxa (18% of the gamma diversity) was observed in the SHD treatment peaking in the SA for bacteria. Benthic macroinvertebrates and bacteria shared a generally low number of taxa across treatments and locations; approximately, 25% of the gamma diversity was shared among all treatments and locations for macrofauna, dropping to 11% for bacteria. Given the low overlap in the species distribution across the lagoon, sustaining the connectivity among heterogeneous soft sediment habitats appears to be essential for maintaining regional biodiversity. This study addresses a current scientific gap related to the relative contributions of vegetated and unvegetated habitats to biodiversity in tropical regions.

## Introduction

In tropical coastal waters, seagrass beds, mangroves and coral reefs are often linked through physical, chemical and biological processes^1–4^. These interconnected habitats form what is often referred to as “the tropical seascape”^5^, which contributes to a variety of ecosystem goods and services essential for human well-being^2,6,7^.

Among the typical triad of tropical key marine habitats, seagrass meadows are considered one of the most productive^8,9^. Their presence increases habitat complexity and ecological niches^10–12^, enhancing biodiversity. By reducing current velocity and wave action, seagrasses promote organic and inorganic matter deposition^13,14^. Both factors are hypothesized to favor the presence of more macroinvertebrate species and/or individuals within seagrass beds which can ultimately result in higher prey availability for economically important fishes compared to nearby unvegetated areas. Seagrass habitats also provide shelter from predators and can act as juvenile nursery grounds. Additionally, many charismatic marine species such as dugongs, manatees and sea turtles depend directly on these habitats for food^8,15–18^. Seagrasses, therefore, represent priority habitats for conservation^19^. Nevertheless, they have been severely affected worldwide, with an estimated loss in cover of 110 km^2^ yr^−1^ since 1980^20^.

While seagrass meadows can be naturally fragmented by processes such as extreme weather events, waves and currents^21,22^, anthropogenic pressures including coastal construction, eutrophication, dredging and infilling, trawling, sewage discharges and recreational activities^22–26^ have greatly increased their fragmentation. Fragmentation in seagrass cover is likely to impact faunal assemblages^27–29^, with exclusive species usually being the most vulnerable to habitat change^30^. Considering the functional connections across the tropical seascape, impacts of fragmentation have the potential to spread beyond the directly impacted local area and could alter patterns of ecological connectivity among coastal habitats^4,31,32^ and food webs^33^, affecting ecosystem resilience and functioning. Few studies have, however, addressed the impacts of seagrass fragmentation on the structure and functioning of their associated biological communities in tropical and subtropical environments^19,34,35^. Furthermore, studies on the effects of seagrass cover usually focus on a single biological component, mainly fish^36^ or macroinvertebrates^27^, and to a lesser extent a combination of both components^37,38^. While pelagic-benthic coupling has been recognized as an important process in shallow environments, especially in areas such as coastal lagoons with limited water turnover^39^, there are limited studies that evaluate both processes together.

Compared with macrofauna, the contribution of microbes (planktonic, epiphytic or benthic) to biogeochemical cycling in seagrass meadows has received little attention to date. However, microbial communities are associated with a variety of key functions including nitrogen fixation^40^, nutrient recycling^41^ and decomposition of organic matter^42^ from both autochthonous and allochthonous sources. Indeed, much of the particulate primary production in seagrass meadows is unavailable to animal consumers and becomes detritus^43^. Furthermore, a substantial fraction of photosynthate is released as dissolved compounds thus contributing to dissolved organic matter (DOM) stocks^44^, the major organic matter and energy source for free-living heterotrophic prokaryotes (mostly bacteria, since Archaea are rare in shallow ecosystems). Release of bioavailable DOM occurs not only through seagrass leaves but also through their roots thus feeding both pelagic and benthic heterotrophic prokaryotes/bacteria^45,46^. Their high metabolic rates, provide a vital link in the transfer of bioavailable DOM to higher trophic levels, supporting the rich benthic food webs^43,47^. Macrofauna also play a significant role in nutrient cycling^48^. Macroinvertebrates including crustaceans, gastropods, bivalves, polychaetes and echinoderms have been implicated in: (1) structuring the benthic environment through processes such as bioturbation and burrow construction^48–51^; (2) aeration of the sediment fostering aerobic bacterial activity^52,53^; and (3) acting as food sources for higher trophic levels including many ecologically and commercially important fish species^54^. Despite the clear linkages between macroinvertebrates and microbes, these two fundamental biological components of seagrass biotopes are rarely analyzed together.

This study therefore aims to investigate the potential effects of seagrass density on tropical soft sediment communities using a coastal shallow lagoon of the central Red Sea as a case study. While representing one of the first attempts at combining the responses of benthic macroinvertebrates and bacteria it also contributes to understanding the ecological consequences of seagrass fragmentation in tropical regions, where knowledge is still limited. We use different densities of seagrass as a proxy for fragmentation, ranging from high density of vegetation to unvegetated bottoms. We characterized and then compared benthic communities across four density treatments in three subtidal lagoon locations. The two main questions addressed are: (1) Do benthic communities of macroinvertebrates and bacteria change across seagrass density gradients? (2) What are the main drivers of the observed patterns? This information is vital to determine whether spatial properties of vegetated marine habitats influence soft sediment diversity, with potentially important implications for managing coastal habitats.

## Material and methods

### Study site

The present study was conducted at the Al Qadimah lagoon (22° 22′ 39.3″ N 39°, 07′ 47.2″ E; approximate area of 13.9 km^2^), located in the central part of the western coast of the Saudi Arabian Red Sea (18°–25°N), where the highest seagrass diversity is observed^55^. The lagoon is dominated by shallow subtidal sands (average depth 2.19 m) with a maximum depth of approximately 10 m in the channel that connects the lagoon to the open sea. This lagoon does not experience direct anthropogenic disturbances typical of other coastal lagoons (e.g. sewage discharges, fisheries practices, habitat destruction for coastal development). Riverine inputs are also non-existent. However, it is located between two urbanized areas developed over the last two decades (KAUST, King Abdullah University of Science and Technology, 7,000 inhabitants; KAEC, King Abdullah Economic City, < 5,000 inhabitants but it is expected to reach 50,000 over the next years). Despite the lack of direct anthropogenic influences, recent studies^56^ quantified moderate levels of metals in the seagrass meadows.

The bottom of the lagoon, particularly in the inner sheltered areas, is covered by seagrass meadows often consisting of at least two species. The areas are mostly dominated by *Cymodocea rotundata*, *Cymodocea serrulata* and *Enhalus acoroides*, sometimes with algae interspersed. In general, multispecies meadows are dominant. Therefore, the selection of seagrass areas was not limited by the species composition. Previous studies in tropical areas did not find an effect of the vegetation type on densities, species richness or even composition of epifaunal and infaunal communities^19^.

### Sampling design and strategy

**Figure 1:**
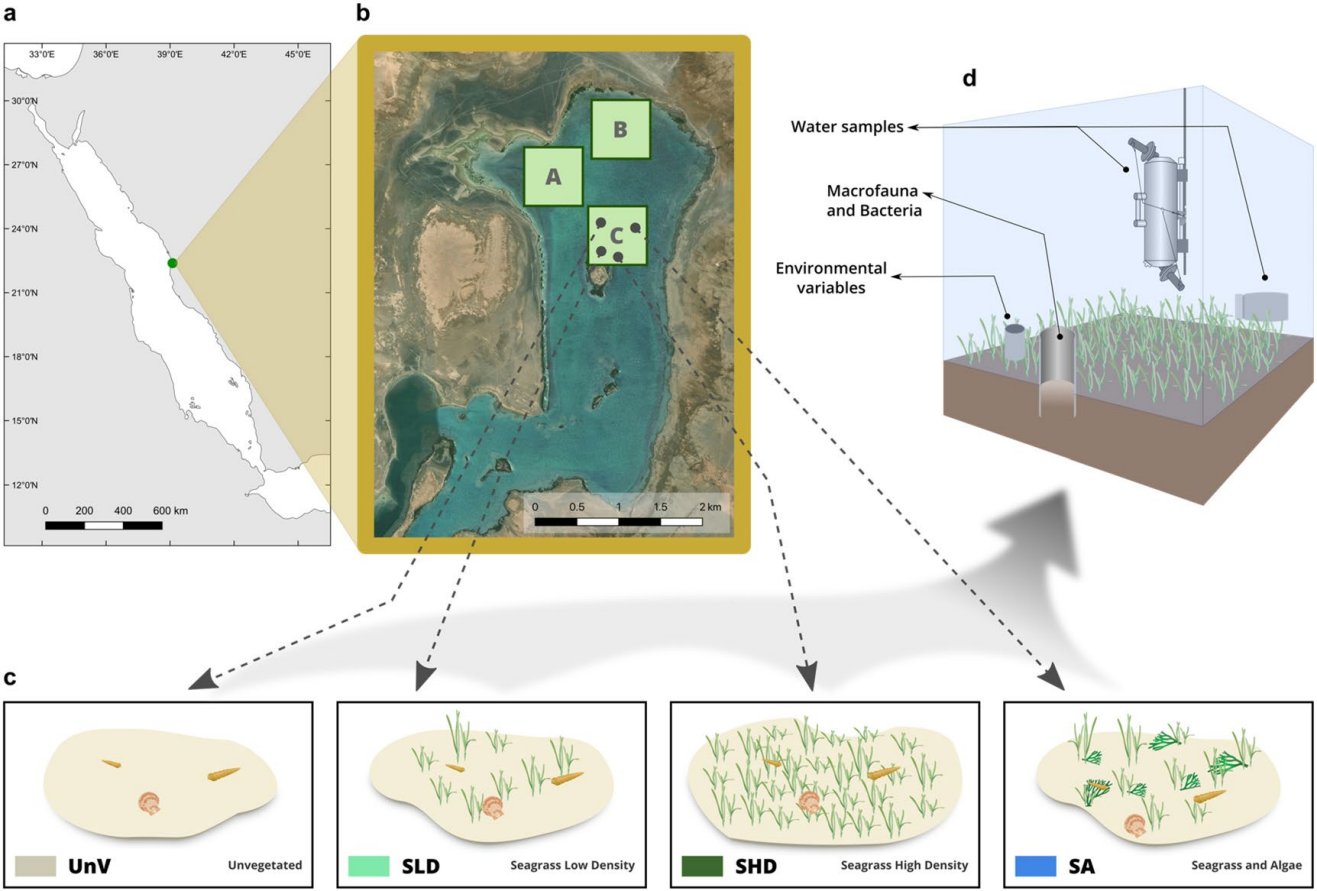
Map showing the location of the Al Qadimah lagoon, the three locations where samples were collected (**a**, **b**), as well as an illustration of the different treatments (**c**) and sampling strategy (**d**). Unvegetated (UnV); Seagrass Low Density (SLD); Seagrass High Density (SHD); Seagrass and Algae (SA). A, B, and C denote the three locations. (**a**) The map was created in QGIS Geographic Information System. Open Source Geospatial Foundation Project. https://qgis.org version 3.10.4—A Coruña. (**b**) satellite imagery from: Esri, “World Imagery" [basemap], https://services.arcgisonline.com/ArcGIS/rest/services/World_Imagery/MapServer, (April 5, 2020). ClipArts of benthic organisms represented (**c**) from IAN Image Library (ian.umces.edu/imagelibrary).

The survey was conducted in naturally fragmented seagrass beds in March 2017. Considering the lack of knowledge on the hydrodynamics of the lagoon, three locations were randomly selected (A, B, C; Fig. 1), to assess whether the location of the meadows would affect the response of microbial and macrobenthic benthic communities to habitat fragmentation. All sampling sites were surveyed within a depth range of 1.6–2.8 m.

In each location, four different treatments were established. These are representative of what is commonly observed in the lagoon but also of different alternative states associated with seagrass fragmentation. The treatments were established based on the seagrass density: unvegetated (UnV) with estimated percentage cover of 0–5%; seagrass low density (SLD) with estimated percentage cover of 10–25%; high density (SHD) with estimated percentage cover of 50–75%; and seagrass and algae (SA) mixed meadows. In the field, triplicate quadrats (50 × 50 cm) were placed at random within each treatment in order to obtain the percentage of seagrass and algae cover (%) and to measure shoot density (m^2^).

A combination of sediment and water samples was collected at each location and treatment, following a block sampling design with two factors: Location (fixed, three levels: A, B and C) and Treatment (fixed, four levels: UnV, SA, SLD, SHD).

#### Water samples

At each sampling site, CTD casts were carried out for conductivity, temperature and depth (CTD) with a multiparameter probe (OCEAN SEVEN 316 Plus and 305 Plus CTD). Using Niskin bottles (5 L), water samples were collected at each location. Divers, using 1 L bottles, also collected samples a few centimeters above the bottom while avoiding sediment disturbance (overlaying water samples). For nutrients, triplicate 50 mL of water (both in the water column and overlaying water) were filtered on the boat (Isopore membrane filters, 0.2 μm GTTP) and then stored in the dark and cool until they were frozen at − 50 °C in the lab. For chlorophyll *a* (Chl *a*), water samples were later filtered in the lab through 47 mm GF/F filters, wrapped in aluminum foil and preserved at − 80 °C until extraction of the pigments. For dissolved organic carbon (DOC) and total dissolved nitrogen (TDN) concentrations, water was filtered through pre-combusted GF/F filters, acidified with phosphoric acid 87% down to pH 1–2 and stored at 4 °C until analysis. For fluorescent dissolved organic matter (FDOM) analysis, water was filtered through pre-combusted GF/F filters, kept at 4 °C and analyzed within 6 h of collection.

#### Sediment samples

In each location and treatment, three replicate core samples were taken (12 cm internal diameter, 20 cm depth) for the study of macroinvertebrates. Each sample was composed of three cores (total area of 0.03 m^2^); 36 replicates in total were collected. In order to avoid edge effects typical of seagrass meadows^57^, samples were whenever possible taken from the innermost area of each treatment. In the field, sediment was washed over a 1 mm sieve, and samples were preserved in ethanol (96%). For benthic bacterial analyses, 5 g of sediment was taken in duplicates. These 5 g samples were placed in RNA later, and frozen until further laboratory sampling. Additional sediment cores were collected to quantify associated environmental variables, specifically, total organic carbon (TOC), grain-particle size, and nutrients in pore-water (NO_3_^−^, NO_2_^−^, PO_4_^3−^ and SiO_4_^2−^).

### Laboratory analyses

#### Water samples

Nutrients (dissolved nitrate (NO_3_^−^), nitrite (NO_2_^−^), phosphorus (PO_4_^3−^), and silicates (SiO_4_^2−^) were quantified using a Continuous Flow Analyzer (SEAL AutoAnalyser 3 with XY2/3 Sampler). To quantify nutrients in pore water samples, sediment was centrifuged for 30 min at 3,000 rpm to separate the pore water from the solid fraction.

Chl *a* quantification was undertaken using the Acetone (10 mL at 90%) extraction method. Briefly, samples were left in acetone for 24 h in low light conditions to minimize degradation. A Turner Trilogy fluorometer (Turner Designs) was used to quantify the Chl *a* content using an acidic module. The degradation of the Chl *a* to phaeophytin was accomplished by acidifying the sample with 60 µL of 0.1 N HCl. DOC and TDN measurements from the water column were performed by high temperature catalytic oxidation (HTCO) using a Shimadzu TOC-L. To monitor the accuracy of the estimated carbon, we used reference material of deep-sea carbon (42–45 μmol C L^−1^ and 31–33 μmol N L^−1^) and low carbon water (1–2 μmol C L^−1^). Dissolved organic nitrogen (DON) concentrations were calculated after subtracting the dissolved inorganic nitrogen (DIN) to the TDN (DON = TDN − DIN), where DIN (μmol N L^−1^) = [NO_3_^−^] + [NO_2_^−^], and DOM C:N ratios were calculated by dividing DOC by DON. FDOM analysis were performed using a HORIBA Jobin Yvon AquaLog spectrofluorometer with a 1 cm path length quartz cuvette. The three-dimensional fluorescence excitation emission matrices (EEMs) were recorded following the same steps as in Calleja et al.^58^. The EEMs were modeled together with other FDOM samples (> 400) collected at the same lagoon within a time series study conducted between 2015 and 2017 (unpublished data). A four-component model was validated using split-half validation and random initialization^59^ and the following peaks were identified: peak C1 at Ex/Em 252/ 397 nm (humic-like peak A^60^), peak C2 at Ex/Em 252/467 nm (a combination of humic-like peaks M and C^60^ peak C3 at Ex/Em 303/337 (protein-like peak T^60^ and attributed to Tryptophane), and peak C4 at Ex/Em 270/312 nm (protein-like peak B^60^ and attributed to Tyrosine). The maximum fluorescence (Fmax) of each component is reported in Raman units (RU). Autotrophic and heterotrophic picoplankton abundance in the water column samples was measured by flow cytometry using standard protocols described in detail in Gasol and Morán^61^ and Silva et al.^62^. Briefly, 1.8 mL samples were fixed with 1% paraformaldehyde + 0.05% glutaraldehyde, deep frozen in liquid nitrogen and stored at − 80 °C until analysis. After samples were thawed, 0.4–0.6 mL aliquots were run through a FACSCanto II flow cytometer (BD Sciences) to determine the abundance of cyanobacteria and heterotrophic prokaryotes. Only *Synechococcus* were detected among the latter since *Prochlorococcus* avoid shallow, enclosed environments such as the lagoon^62^. The widespread groups of low and high nucleic acid content of heterotrophic prokaryotes were distinguished based on the relative green fluorescence after staining the samples with SybrGreen (Molecular Probes). Flow rates were determined empirically to obtain cell abundances.

#### Sediment samples

These samples were dried at 60 °C until constant weight prior to their homogenization in an automatic mill grinder (model PM200 from Retsch) where the samples were milled for 4 min at 420 rpm. Afterward, the fine powder was collected and prepared for analyses. Total carbon and nitrogen were determined for the powder samples. Inorganic carbon and nitrogen fractions were also calculated after loss on ignition (450 °C for 3 h). Analysis was performed in a CHNS-O “Thermo Scientific” organic elemental analyzer (model Flash 2000), with soil standard 2G NCS as reference material. Particulate and total sediment organic carbon (POC and TOC, respectively) and nitrogen (PON and TON, respectively) were calculated by the difference between the total and inorganic fractions. For trace element analysis (Al, Ba, Cd, Co, Cr, Cu, Fe, Pb, Mg, Mn, Ni, Se, V, and Zn), samples were dried at 40 °C and 0.2 g was analyzed by flame atomic absorption spectrophotometer AAS (Perkin Elmer, Model 2380A Spectrophotometer) following the EPA 2007 methods, as detailed in^56^. Particle size analysis was conducted by wet separation of the silt and clay fractions from the sandy fractions. Replicates of sediment samples were sieved through a 63 μm mesh (silt and clay fraction), and the retained sediment fraction was dried at 80 °C for 24–48 h. The dried sample material was sieved by using a column of sieves. Fractions were classified as: gravel (> 2 mm), very coarse sand (> 1 mm and < 2 mm), coarse sand (> 500 μm and < 1 mm), medium sand (> 250 μm and < 500 μm), fine sand (> 125 μm and < 250 μm), very fine sand (> 63 μm and < 125 μm), and silt–clay (fines, < 63 μm). Seagrass material (roots and leaves) were separated and biomass measured as dried weight.

#### Benthic macrofauna

Samples were sorted and organisms identified to the highest taxonomic separation level (50% of OTUs at the species level) and counted. The lack of taxonomic knowledge for most of the soft-sediment invertebrates in the region, together with the preservation of samples in ethanol precluded the identification of organisms to the same taxonomic level.

#### Benthic bacteria

Samples were processed using the PowerMax Soil DNA isolation kit (Qiagen) as per the manufacturer's instructions with the exception of step 3 where the bead beating was replaced by the addition of 0.4 mg mL^−1^ of Proteinase K and an overnight incubation in a shaking incubator at 56°C^63^. The bacterial component was amplified with PCR by targeting the v3 and v4 regions of the 16S rRNA gene using the primers (341F: 5′-CCTACGGGNGGCWGCAG-3′ and 805R: 5′-GACTACHVGGGTATCTAATCC-3′^64^. The reaction mixture for the 16S rDNA was held at 94 °C for 5 min followed by 30 cycles of 95 °C for 15 s, 52 °C for 15 s, 72 °C for 15 s, with a final extension step at 72 °C for 7 min. A negative PCR control was run at the same time as the sample PCR’s. The triplicate PCR products from each sample were pooled, cleaned and normalized using SequelPrep Normalization plates (ThermoFisher Scientific). After cleaning and normalization, Illumina tags were added via a second round of PCR amplification using the Illumina 16S metagenomic sequencing library preparation protocol (https://support.illumina.com/documents/documentation/chemistry_documentation/16s/16s-metagenomic-library-prep-guide-15044223-b.pdf) and, subsequently cleaned and normalized using the SequelPrep Normalization plates (ThermoFisher Scientific). An Illumina MiSeq sequencing platform (v3 chemistry) at the King Abdullah University of Science and Technology (KAUST) Bioscience Core Laboratory (BCL) was used to generate the sequences (2 × 300 bp). Raw reads were deposited at the NCBI Short Read Archive under the project accession PRJNA592571.

### Data visualization and statistical analyses

#### Environmental data

Principal Component Analysis (PCA) was used to create an ordination plot of the samples based on the environmental variables that were measured. Prior to the analysis, the correlation between variables was assessed (Pearson R). Highly correlated variables (> 0.7) were excluded, which primarily related to the concentration of metals and grain size.

When correlation between two variables was higher than 0.7 only one of the variables was chosen based on the following criteria: (1) the one that was less correlated to other variables; (2) the explanatory importance/potential of the variable for the specific case; (3) the extension of the gradient (i.e., variables with a larger gradient (range) were preferred to small gradients). Correlations between 0.5 and 0.7 were also subjected to the same procedure to further reduce the number of correlated variables in the analysis. Finally, 10 variables were used in the PCA analysis (fines, TOC, DOC, Chl *a*, TDN, NO_2_^−^_pw_, PO_4_^3−^_pw_, NO_2_^−^ and NO_3_^−^). Information regarding the multiple correlations between variables is provided in Supplementary Material (S1). Variables that did not present a normal distribution were transformed (log_e_ or Box-Cox transformation). Environmental data was normalized by scaling each variable to zero mean and unit variance before the analyses using function “decostand” from R package “vegan”^65^.

#### Macrofauna

Several univariate metrics were calculated including density (ind. m^−2^), and the average number of taxa (i.e., number of taxa × 0.03 m^−2^). Two-way ANOVAS were conducted for the factors *Treatment* and *Location* after checking for normality and homogeneity of the data. Normality was checked using Shapiro–Wilk normality test and by using quantile–quantile plots. Homogeneity of variance was tested using Levene's test. Whenever normality or homogeneity of variance was not verified the data was transformed (log_e_) to meet ANOVA assumptions. Post-hoc tests (Tukey Honest significance test) for ANOVA significant factors were used to ascertain for differences between groups of samples. Community structure was investigated by ordination techniques (Principal Coordinate Analysis), based on the Bray–Curtis dissimilarity coefficient applied to untransformed abundance data as the dataset is characterized by low abundance values and there is no need to reduce the weight of the high abundance taxa. Permutational multivariate analysis of variance (PERMANOVA), for the two-factors in analysis (i.e. *Treatment* and *Location*) was applied to assess for significant differences in the associated fauna. When significant differences were detected, pair-wise tests were applied. Distance-based redundancy analysis (dbRDA) was also applied in order to understand the relationship between each environmental variable and benthic community structure (given by the direction and length of vectors for each variable). A set of 16 variables was selected from the original 37 to reduce collinearity problems using the criteria used for the PCA but only removing the variables that were highly correlated in each domain. The set comprised grain size (fines and coarse sand), dissolved inorganic nutrients (NO_2_^−^, NO_3_^−^, PO_4_^3−^, NO_2_^−^_pw_, NO_3_^−^_pw_, PO_4_^3−^_pw_), trace elements (Cu, Pb, As), productivity proxies (Chl *a*) and other nutrients (TOC, DOC, TDN) and seagrass density (density of seagrass shoots). Due to differences in the scales and units of environmental variables, environmental data was normalized by scaling each variable to zero mean and unit variance before the analyses. The model used for the dbRDA was manually built to maximize the explanatory power of the variables, starting from a model with no explanatory variables and adding or removing variables according to their capability to further explain the variability of the community data (i.e., evaluating all single terms that can be added or dropped from the constrained ordination model using “add1.cca” and “drop1.cca” functions; R package “*vegan*”^65^). The selection of the variables was based on statistics (F and AIC for the single terms) but also considering the ecological processes (several processes instead of a set of variables related to one process). The significance of each variable retained in the model was assessed using marginal and sequential tests.

All analyses were undertaken in R^66^ with the following packages: PCA, “*FactoMineR*”^67^; PCoA, “*ecodist*”^68^; dbRDA, “*vegan*”^65^; “*VennDiagram*”^69^, except for the PERMANOVA and SIMPER analysis that were conducted using Primer v7^70^.

*Benthic bacteria*. The “*dada2*” package^71^ was used to process the raw sequencing reads within R^66^. The raw reads were truncated based on quality and then filtered with a maxEE of 2 for forward reads and 6 for reverse. Error rates were calculated for the filtered reads and subsequently paired ends were merged with a maximum mismatch of 1 and a minimum overlap of 10. The function removeBimeraDenovo was used to remove chimeras and taxonomy was assigned to each representative sequence against the Silva v128 database^72^ within the “*dada2*” package resulting in a table of operational taxonomic units (OTUs) against sample, using the “*phyloseq*” package^73^. Any sequences assigned as eukaryotes and Archaea, as well as chloroplasts and mitochondria were subsequently removed bioinformatically. This removed 6.2% of OTUs but only 0.48% of reads. Samples were rarefied to an even depth (10,000 reads) for comparison. Samples not meeting this criterion were removed from analysis. After the creation of the OTU table, the same statistical analytical routines were undertaken as described for the macrofauna. Multivariate analysis was based on the log-transformed data to down weight the weighting of taxa with a high number of sequences. A two-way ANOVA was conducted to test for differences regarding *Treatment* and *Location* (see previous section “Macrofauna”). Due to the unbalanced design a Type III ANOVA was used on transformed values (log_e_).

### Responses of taxa to habitat drivers

A forward step-wise regression model using the Bayesian Information Criterion (BIC) exit criterion was used to determine the environmental parameters that explained benthic taxa differences between treatments. The taxa identified as important contributors for the biological patterns based on Similarity Percentage (SIMPER) analysis (Primer v7) were analyzed using step-wise regression. Regression models that account for a large number of potentially relevant variables may lead to over-fitting and poor prediction performance. Hence, environmental variables excluding those that were highly correlated were carried forward in the regression. Linear regression models were then applied to identify the magnitude and direction effect of the important environmental variables identified from the stepwise regression that significantly contributed to benthic abundance data.

## Results

### Environmental variability among locations and treatments

**Figure 2:**
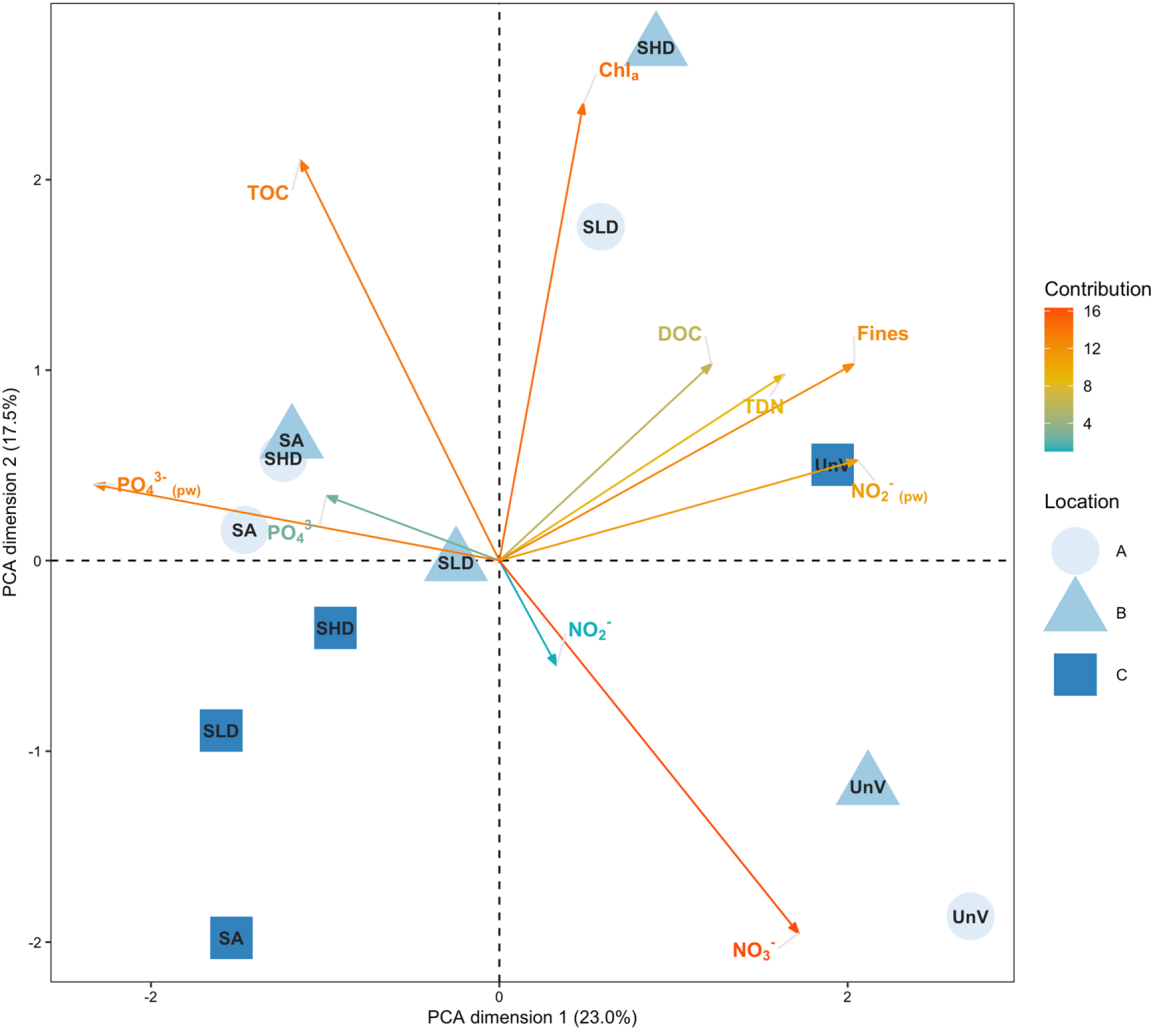
Principal Components Analysis (PCA) ordination plot of a subset of environmental variables across treatments (Unvegetated, UnV; seagrass and algae, SA; seagrass low density, SLD; seagrass high density, SHD) and locations (circles, A; triangles, B; squares, C). Information regarding multiple correlations between variables is provided in supplementary material. Fines, sediment fraction < 63 μm; DOC, dissolved organic carbon; TDN, total dissolved nitrogen; Chl *a*, chlorophyll *a*; TOC, total organic carbon; pw denotes dissolved inorganic nutrients quantified in sediment pore water. The number on each axis indicates the percent variability explained by that principal component. The color scale and the length of each environmental vector are related to the contribution to the total variance.

The PCA diagram shows a general separation of the unvegetated samples (UnV, lower right quadrant) from the rest of the samples. Samples from location C mainly cluster in the left lower quadrants. The first two dimensions (Axis 1: Fines, PO_4_^3−^_(pw)_, NO_2_^−^_(pw)_, DOC, TDN and PO_4_^3−^; Axis 2: Chl *a*, TOC, NO_3_^−^, and NO_2_^−^) of the PCA analysis explain 40.5% of the variability for the selected environmental variables (Fig. 2).

Important variables contributing to differences between samples were related to grain size (fines), dissolved inorganic nutrients (NO_3_^−^ and PO_4_^3−^_pw_), productivity and other nutrients (Chl *a*, TOC, TDN, DOC). Higher values of fines, DOC, TDN and nitrite in the pore water are associated to samples from unvegetated areas whereas vegetated areas presented sediment with higher content of phosphate. Chlorophyll *a* and TOC (and variables correlated to these) separate vegetated samples from Location C from those locations A and B. Within locations A and B, nutrients in the porewater (silicates, phosphates and nitrates), TOC and Chl *a* presented higher values in vegetated compared with the unvegetated treatments.

### DOC, FDOM and autotrophic and heterotrophic bacterioplankton in the water column

DOC concentration and C:N ratios averaged (± SD) 108 ± 4 µmol C L^−1^ and 13.7 ± 1.4, respectively, and did not show significant differences between locations or treatments. Although changes were small, C:N ratios significantly increased with NO^3−^ concentrations (*p* < 0.05), indicating that NO^3−^ depleted samples (due to less production and/or higher consumption) presented DOM richer in nitrogen and vice-versa, i.e., samples with higher NO^3−^ in the water presented DOM richer in carbon. The fluorescent characteristics of DOM identified two humic-like components (C1 and C2), and two protein-like components (C3 and C4). C1 and C2 were always more abundant (range 0.032–0.045 R.U.) than C3 and C4 (range 0.014–0.021 R.U.). The humic fraction ranged between 66 and 69% and did not show significant differences between locations or treatments. However, when correlating the FDOM data to environmental parameters we observed that all components except C4 significantly increased with salinity, despite the limited variability (41.7–42.2). The correlation was particularly strong for the humic components (C1 *p* < 0.001, r = 0.89; C2: *p* < 0.001, r = 0.85) and weaker but still significant for the Tryptophane-like peak (C3: *p* < 0.05 r = 0.70). Humic components also showed a positive and significant relationship with Chl *a* values (C1: *p* < 0.001, r = 0.77, C2: *p* < 0.001, r = 0.80) although no relationship was found with the protein-like components.

Autotrophic bacterioplankton was represented by *Synechococcus*, which ranged from 0.55 to 9.33 × 10^4^ cells mL^−1^. Heterotrophic planktonic bacteria were one order of magnitude more abundant (0.58 to 3.22 × 10^5^ cells mL^−1^) than autotrophs in the pelagic environment. A trend of highest abundance of bacterioplankton in the SLD and lowest in the SHD, with similar intermediate values in unvegetated and mixed meadows was found. Indeed, the abundance of autotrophic and heterotrophic bacteria were strongly correlated (r = 0.93, *p* < 0.001, n = 10). While differences between the four treatments were not significant, abundances of both autotrophic and heterotrophic planktonic bacteria at location B were significantly lower (ANOVA, *p* < 0.05) than at the other two locations.

### Major patterns in benthic diversity and structure

A total of 78 macrobenthic taxa and 920 individuals were identified belonging to 58 families. The most abundant phyla were Annelida (39%), Mollusca (25%), Sipuncula (22%), and Arthropoda (9%), followed by Echinodermata (4%). Total abundance ranged from 156 (unvegetated) to 323 individuals in SLD. For benthic bacteria there were 13,899 OTUs representing 201 families. The dominant phyla in the bacterial community were Proteobacteria (65.6%), Chloroflexi (7.6%), Acidiobacteria (6.1%) and Bacteroidetes (4.1%).

**Figure 3:**
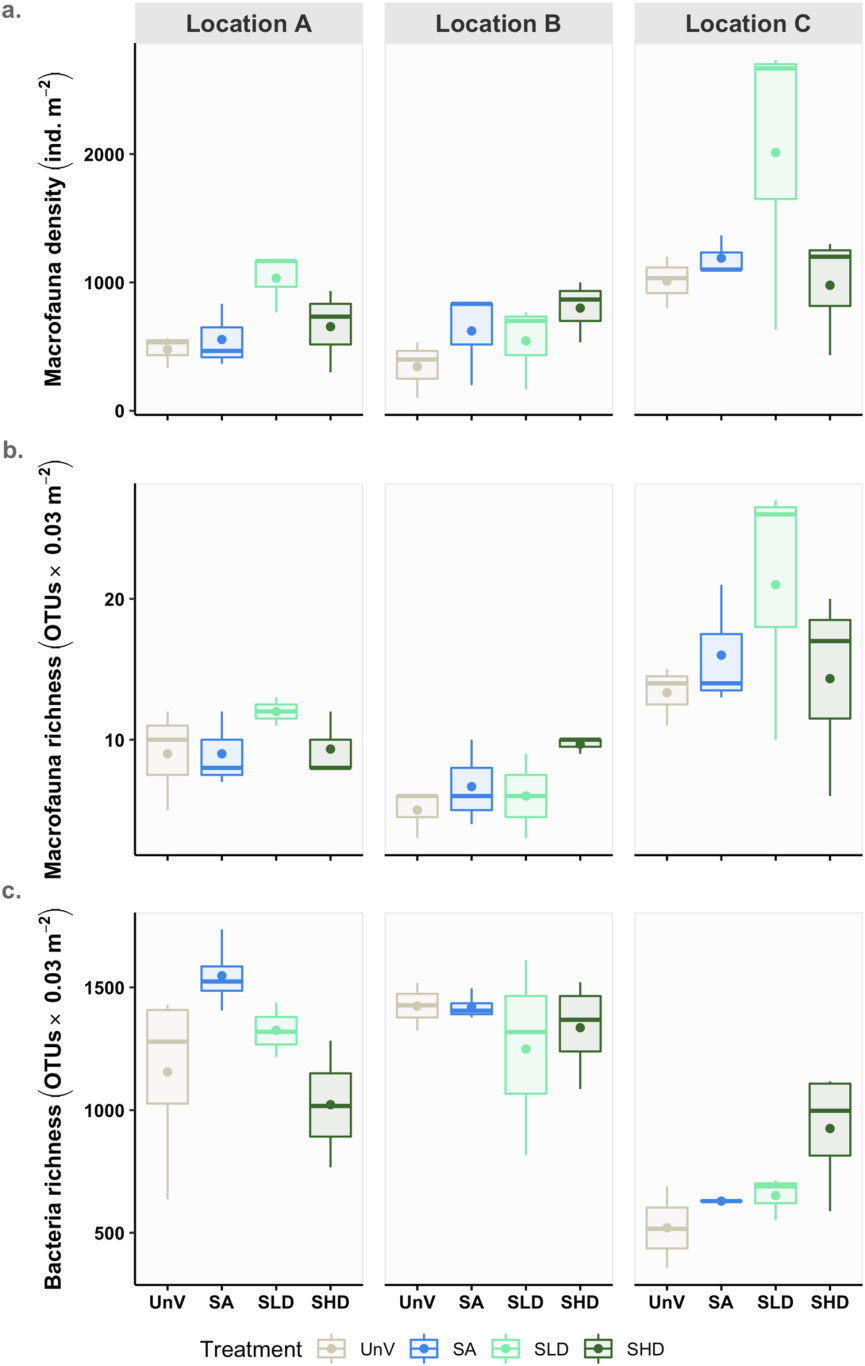
Macrofaunal variability in (**a**) density, and (**b**) richness, and (**c**) variability in bacterial communities richness across treatments and locations. A, B, and C denote the three locations. Unvegetated (UnV); seagrass and algae (SA) mixed meadows; seagrass low density (SLD); seagrass high density (SHD). The dots referred to the average on the plots.

Overall, benthic macrofaunal density (log_e_ transformed data) and number of taxa (log_e_ transformed data) did not vary significantly among treatments (ANOVA; Density: F = 1.558, *p* = 0.226; Number of OTUs: F = 0.937, *p* = 0.440) but there was a significant effect of *Location* (ANOVA; Density: F = 7.201, *p* = 0.004; Number of OTUs: F = 14.491, *p* < 0.001). Both metrics were significantly higher in location C, compared to locations A and B (Fig. 3A, B). Regarding the number of macrofauna taxa (log_e_ transformed data), significant differences were also observed between locations A and B (Tukey post-hoc test, *p* = 0.044). For bacteria, the number of OTUs was generally higher in locations A and B than in location C (Fig. 3C). The observed number of taxa showed, however, a significant interaction between *Location* and *Treatment* (F = 2.546, *p* = 0.043). Post-hoc tests showed significant differences between locations C and both A and B in all treatments, except SHD (no significant differences). In locations A and B no significant differences were detected between treatments whereas in location C, values in UnV and SHD were significantly different (Tukey post-hoc test, *p* = 0.012) (results in Supplementary Material, Table S1).

**Figure 4:**
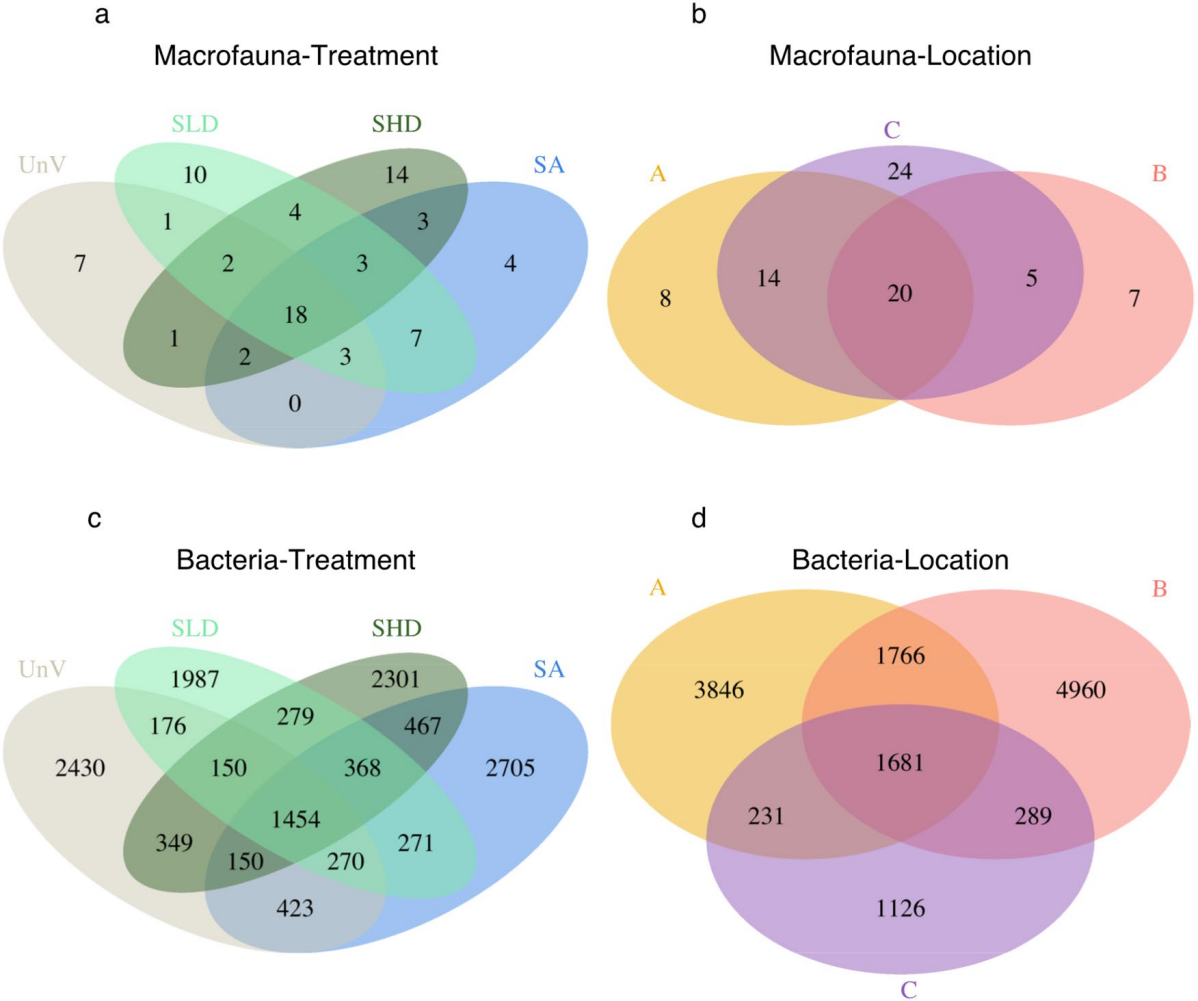
Venn diagram illustrating the numbers of shared and exclusive taxa of macrofauna (**a**, **b**) and bacteria (**c**, **d**) regarding the two factors in analysis, i.e. *Treatment* (**a**, **c**) and *Location* (**b**, **d**). A, B, and C denote the three locations. Unvegetated (UnV); seagrass and algae (SA) mixed meadows; seagrass low density (SLD); seagrass high density (SHD).

A total of 18 macrofaunal taxa representing 23% of gamma diversity (i.e. all taxa identified) were shared among treatments across the three locations. Among the shared taxa, the most abundant was the sipunculid *Phascolion strombus strombus* (20.2% of total abundance). Other main contributors to total abundance were the opportunistic polychaetes Capitellidae und. (7.8%), the bivalves *Barbatia foliata* (7.8%) and *Cardiolucina semperiana* (6.1%), as well as polychaetes from the family Eunicidae (6%). SHD showed the highest percentage of exclusive taxa (18% of gamma-diversity), twice as much as the unvegetated treatment and 3.5 times more than the mixed meadow (Fig. 4). Regarding the factor *Location*, 20 taxa were present at the three locations, with location C showing the highest percentage of exclusive taxa (i.e., 24; 31% of gamma diversity) (Fig. 4a, b).

A total of 1,454 bacteria OTUs were shared amongst all treatments, representing 10.5% of gamma diversity (yet accounting for 78.6% of the relative abundance, Fig. 4c, d). The majority of the abundance in these shared OTUs was classified as belonging to the families Desulfobacteraceae and Syntrophobacteraceae. The mixed meadow (SA) treatment showed the highest number of exclusive taxa (19.3%). Location C showed the lowest percentage of exclusive taxa (8%) with the highest percentage being observed in location B (35%). A total of 62.1% of gamma diversity was observed in location B. This was higher than location A (53.7% of gamma diversity) and location C (23.8% of gamma diversity).

**Figure 5:**
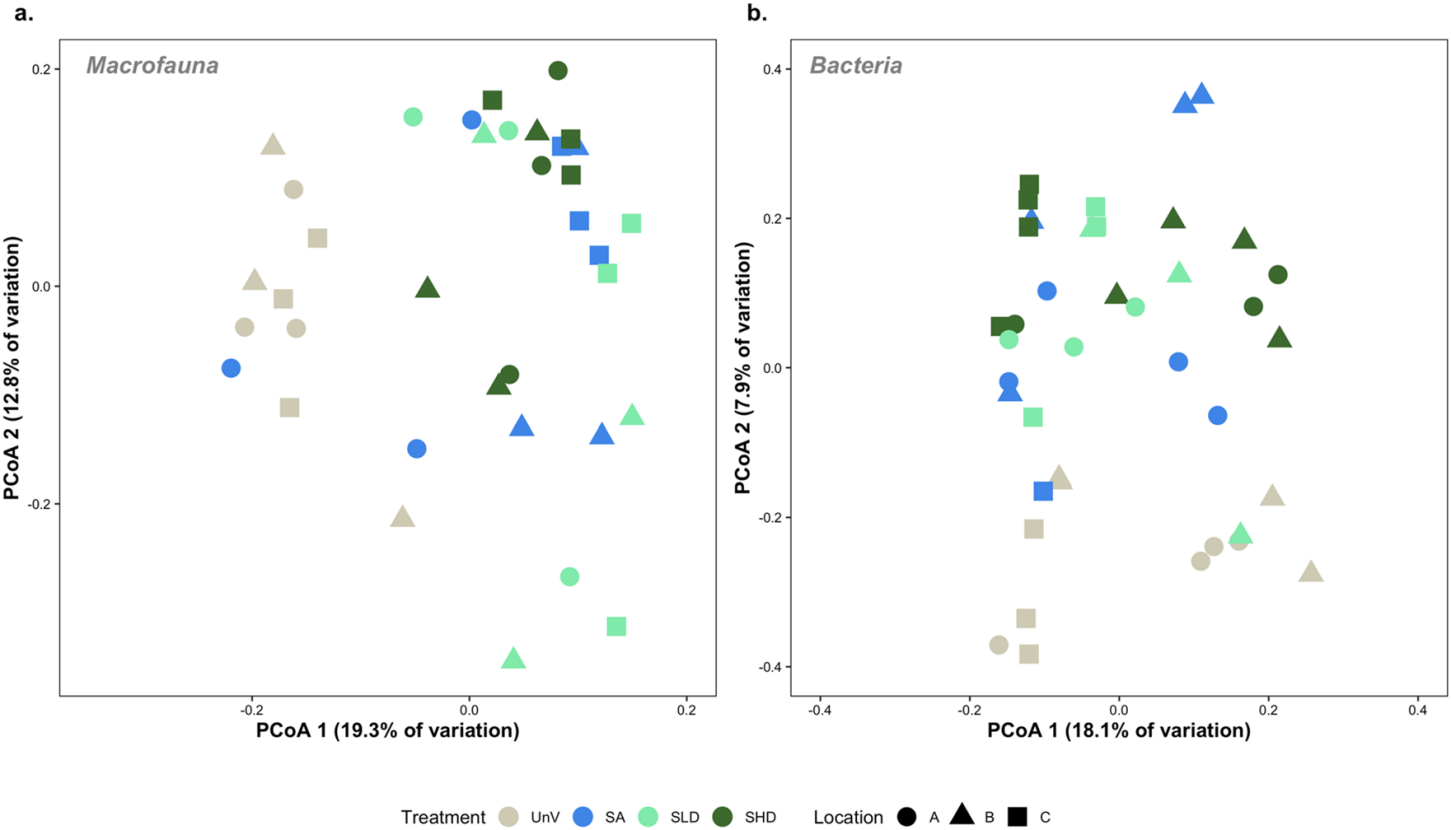
Principal coordinates analysis (PCoA) of macrofauna (**a**) and bacterial communities (**b**) based on Bray–Curtis dissimilarity matrices (bacteria dataset was log_e_ transformed). Samples are colored by treatment type, whereas locations (A, B and C) are represented by symbols. Unvegetated (UnV); seagrass and algae (SA) mixed meadows; seagrass low density (SLD); seagrass high density (SHD).

The principal component analysis (PCoA) showed no clear pattern in the structure of macrofauna assemblages among the treatments with vegetation (i.e., SA, SLD, and SHD) (Fig. 5). However, samples from the unvegetated treatment tended to group separately from other treatments. PERMANOVA analysis showed significant and independent differences in the structure of macrofauna both for *Treatment* (Pseudo-F = 3.1521, *p* = 0.001) and *Location* (Pseudo-F = 2.5591, *p* = 0.001) factors (Table 1; full results presented in Table S2). Pairwise tests showed that assemblages inhabiting unvegetated areas were significantly different from those associated with vegetation (UnV ≠ SA = SLD = SHD). Analysis also showed that location C was significantly different from A and B (*p* < 0.01).

**Table 1:** Two-way PERMANOVA results and pair-wise tests based on Bray–Curtis dissimilarity.

Source	Macrofauna				Bacteria			
	df	MS	Pseudo-F	P(perm)	df	MS	Pseudo-F	P(perm)
**Treatment (Tr)**	3	7041	3.152	**0.001**	3	0.30875	1.788	**0.001**
**Location (Lo)**	2	5716.5	2.559	**0.001**	2	0.40342	2.337	**0.001**
**Tr × Lo**	6	2475	1.108	0.251	6	0.22202	1.286	**0.018**
**Res**	24	2233.7			28	0.17265		
**Total**	35				39			
Pair-wise comparisons								
*Term* ‘*Tr*’					*Term* ‘*Tr × Lo*’			
Groups		t	P(MC)		Within level ‘SHD’ of factor ‘Treatment’			
UnV, SHD		2.312	**0.001**		Groups		t	P(MC)
UnV, SLD		2.270	**0.001**		B, C		1.841	**0.018**
UnV, SA		2.194	**0.002**					
*Term* “*Lo*”					Within level ‘C’ of factor ‘Location’			
Groups		t	P(MC)		Groups		t	P(MC)
A, C		1.705	**0.007**		UnV, SHD		1.7021	**0.038**
B, C		1.726	**0.005**					

Unvegetated (UnV); seagrass and algae (SA) mixed meadows; seagrass low density (SLD); seagrass high density (SHD). A, B and C denote locations. Significant values are shown in bold.

Multivariate patterns of benthic bacterial communities also showed that communities associated with location C were separated along the first axis from those sampled in locations A and B. Samples from the unvegetated habitats were also mainly separated from the remaining treatments on the lower half of the plot (Fig. 5). PERMANOVA analysis identified a significant interaction between *Treatment* and *Location* (Pseudo-F = 1.291, *p* < 0.05). Pair-wise comparisons showed significant differences between locations B and C for the SHD treatment. Also, unvegetated assemblages in location C were significantly different from those associated with the high density (SHD) (Table 1).

### Environmental drivers of benthic communities

**Figure 6:**
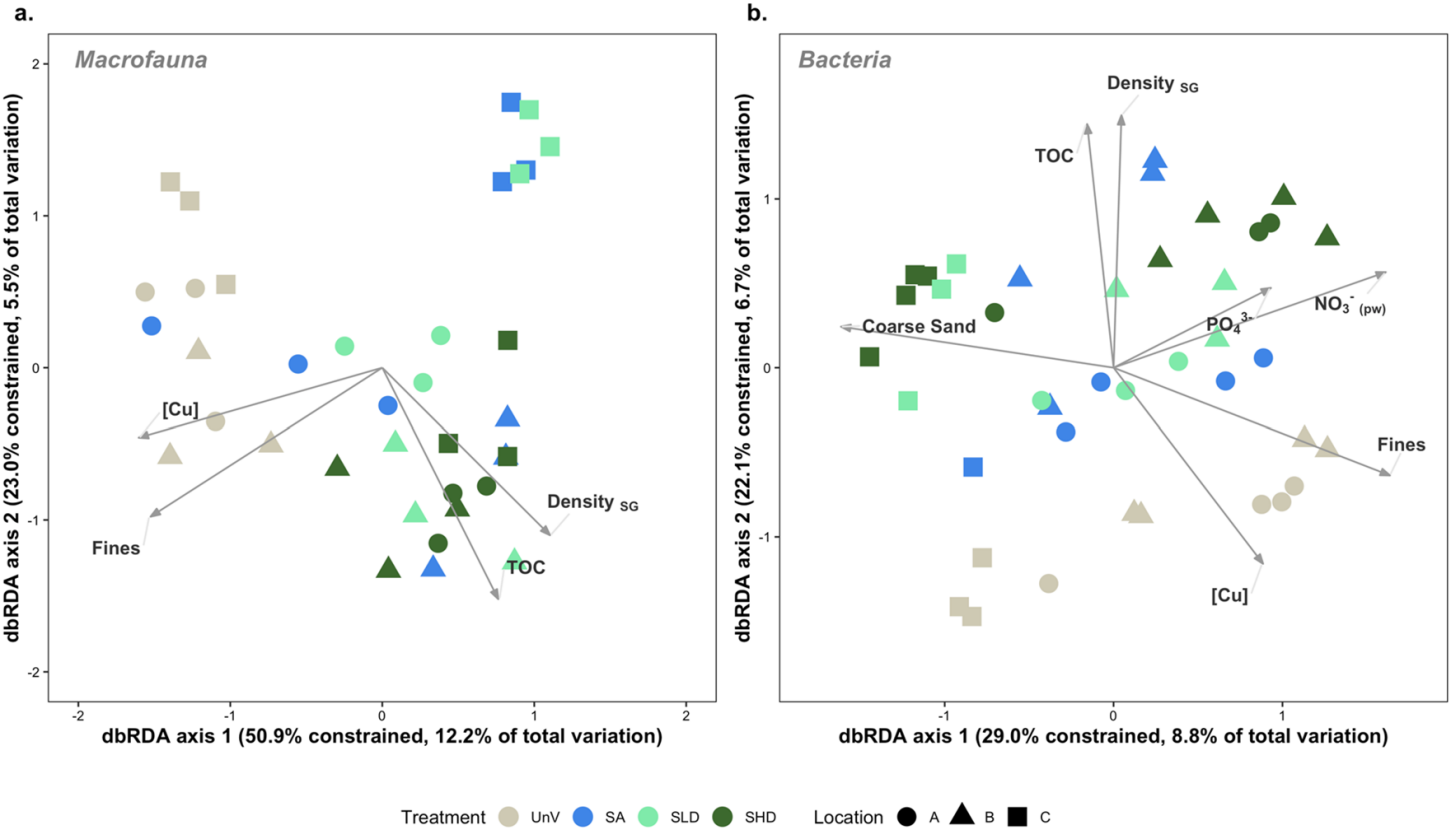
Distance based redundancy analysis (dbRDA) of macrofaunal (**a**) and bacterial (**b**) samples based on Bray–Curtis distance matrices. The dbRDA axes describe the percentage of the fitted or total variation explained by each axis. Samples are colored by treatment type, whereas locations (A, B and C) are represented by symbols. Unvegetated (UnV); seagrass and algae (SA) mixed meadows; seagrass low density (SLD); seagrass high density (SHD). TOC, total organic carbon; Fines, sediment fraction < 63 μm; Coarse Sand, sediment fractions between 0.5 and 1 mm; [Cu], Concentration of trace metal copper in sediments (mg/kg); Density _SG_, Seagrass density; pw denotes dissolved inorganic nutrients quantified in sediment pore water. The length of each environmental vector is related to the contribution to the total variance.

For macrofauna, the dbRDA (Fig. 6a) indicated that the percentage of fine particles in the sediment, seagrass shoot density, total organic carbon and the concentration of Cu (as proxy for most of other trace elements) were the environmental variables significantly driving community composition. The first two axes explained approximately 18% of the total variation. For bacteria (Fig. 6b), along with those variables significant for the macrofauna, nitrate in the pore water (NO_3_^−^_pw_), coarse sand and phosphate (PO_4_^3−^) were also significant. However, a lower percentage of variation was explained (15.5%) compared to macrofauna. The general pattern observed in the PCoA analysis for both communities is also observed here, i.e. the separation of the unvegetated samples from the remaining sites. In both plots, the discrimination between these two communities was mainly due to fine particles in the sediment, concentration of Cu, TOC and shoot density. Nutrients, namely nitrate in the pore water and phosphate seem to be responsible for some separation of the samples from location C regarding the bacterial community composition.

### Responses of taxa to habitat drivers

Taxa selected for regression modeling showed species-specific responses to a combination of seagrass properties, temperature, depth, salinity and grain-size. Taxa that were associated with seagrass habitats, and more specifically that were positively associated with higher seagrass biomass, included the polychaete families Eunicidae, Terebellidae, Capitellidae and Syllidae (Table 2). The brittle star, *Amphiura* sp., was also negatively associated with seagrass beds (notably seagrass shoot density). Conversely, the bivalve *Cardiolucina semperiana* was negatively associated with higher seagrass biomass (Table 2). Eunicidae and *Cardiolucina semperiana* were also negatively associated with higher TOC which is not surprising given TOC was slightly elevated in the seagrass beds which often trap and retain organic matter. Although not very variable, DOC (103–114 µmol C L^−1^) in the overlaying waters followed an opposite pattern, with higher concentrations in the unvegetated samples. A number of macrofaunal taxa were found to be sensitive to increasing metal concentrations in the sediment, with slightly higher concentrations of metals recorded at the unvegetated sites which were also associated with a higher percentage of fines (< 63 µm). For example, the polychaetes Capitellidae, Terebellidae, Eunicidae, and Syllidae were all negatively associated with increasing Ba, Cd and Pb levels (Table 2). For bacterial communities, genera associated with higher seagrass density included *Pelobacter* and *Tistlia*, whereas *Maricurvus*, the environmental clade genus of the family Spirochaetaceae M2PT2-76, and *Candidatus* Omnitrophus showed the opposite response (see Table 2). Yet, M2PT2-76 was associated with higher seagrass biomass. This clade showed significant responses with most of the environmental parameters tested. In particular, it was negatively associated with Ba, Cd, and Pb (Table 2).

**Table 2:** Environmental parameters identified from regression-based analysis to be associated with changes in benthic macrofaunal and bacterial taxa, either increasing (+) or decreasing (−) their abundance.

		D (m)	SST (°C)	S	SG dens	SG biom	TOC	Fines (%)	Vcsand (%)	Gravel (%)	NO_2_ (μM)	PO_4_ (μM)	NO_3_ (μM)	Ba (mg/kg)	Cd (mg/kg)	Pb (mg/kg)	Cu (mg/kg)
**Macrofauna**																	
Class	*Taxon*																
Bivalvia	*Barbatia foliata*	−								+							
Polychaeta	Capitellidae	−			+	+		−	+			+		−			−
Sipunculidea	*Phascolion s. phascolion*																
Polychaeta	Paraonidae																
Polychaeta	Terebellidae			−		+		−	+	+		+		−	−	−	
Malacostraca	Tanaidacea spB											+					
Polychaeta	Eunicidae	−		−		+	−	−	+	+		+		−	−	−	
Bivalvia	*Cardiolucina semperiana*	+			−	−	−	+	−	−	−	−			+		
Sipunculidea	*Apionsoma misakianum*					+		−	+								
Nemertea	Nemertea																
Polychaeta	Goniadidae						−										
Ophiuroidea	*Amphiura* sp	+			−						+	−	−				
Polychaeta	Syllidae	−		−		+		−		+				−	−	−	
Polychaeta	Pilargidae	+				−						−			+	+	
**Bacteria**																	
Flavobacteriia	*Fluviicola*	+	−														
Deltaproteobacteria	*Pelobacter*	−			+		+										
Deltaproteobacteria	*Desulfobacter*										−						
Gammaproteobacteria	*Maricurvus*	+			−							−					
Sphingobacteriia	*Phaeodactylibacter*																
Alphaproteobacteria	*Tistlia*				+		+										
Epsilonproteobacteria	*Arcobacter*																
Spirochaetia	Spirochaetaceae M2PT2-76	−		−	−	+		−	+	+		+		−	−	−	
Clostridia	Defluviitaleaceae										−						
Alphaproteobacteria	*Aestuariivita*																
Gammaproteobacteria	*Kangiella*									+							
Cytophagia	*Fulvivirga*																
Omnitrophica_Incertae_Sedis	*Candidatus Omnitrophus*	+			−						+	−					
Spirochaetes	*Salinispira*																

Only significant (*p* < 0.05) correlations are presented. D, depth; SST, sea surface temperature; Dens. SG, Seagrass density; Biom. SG, Seagrass biomass; TOC, total organic carbon; Fines, sediment fraction < 63 μm; Vcsand, sediment fractions between 1 and 2 mm; Gravel, sediment fractions over 2 mm.

## Discussion

This study provides evidence of the role of seagrass vegetation and local scale environmental processes in influencing benthic community patterns. Here, we have considered both the communities of macroinvertebrates (based on morphological characters) and bacteria (based on 16S rDNA amplicon sequencing approaches) present in the sediment of a Red Sea coastal lagoon. The inclusion of the two endpoints in the size spectrum of benthic heterotrophs (i.e. macroinvertebrates and bacteria) provides a sound basis for the evaluation of the role of seagrass cover as a proxy for habitat fragmentation in an era of escalating degradation of these sensitive habitats^20^. Recent studies have highlighted the utility of benthic bacteria for the assessment of environmental changes using molecular technologies^74^. Assessing responses of both macrofaunal and the microbiome associated with seagrasses may therefore increase our knowledge of the responses of these communities to habitat change.

### Vegetation and environmental conditions drive benthic patterns

We observed that the presence/absence of vegetation (more than its cover) along with changes in local scale sedimentary features (notably grain size, organics and metals) did not significantly influence the amount and characteristics of dissolved organic material in the water column nor did these conditions affect the abundance of free-living planktonic bacteria, including autotrophic and heterotrophic species. We believe that this could be explained by the constant exchange of water between the different sampled locations within the lagoon where the study was conducted. However, the presence/absence of vegetation and different sedimentary features did influence the observed patterns in benthic communities, particularly for macroinvertebrates. Some of the biological parameters analyzed were positively influenced by seagrass density. The high density treatment supported the highest number of exclusive macroinvertebrate taxa (18% of the gamma diversity), twice as much as the exclusive taxa observed in unvegetated areas and more than three times the levels recorded in mixed meadows. These results highlight the role of vegetation, most likely through its secondary effects on local hydrodynamics, food availability and quality, as well as protection from predators, in determining benthic patterns. However, this role was not always consistent across the biological variables analyzed. For example, we did not find unequivocal evidence of a positive correlation between seagrass canopy and the diversity, density and community structure of either bacterial or macrobenthic communities, as responses were site- and taxa-specific. Also, we did not find significant differences between seagrass and mixed meadows containing seagrass and algae.

There is a considerable amount of literature assessing the effects of seagrass canopy on associated assemblages, particularly in temperate regions^75–78^. Less research has investigated differences in benthic assemblages between vegetated and unvegetated substrates within coastal lagoons^79^, and particularly in sub-tropical and tropical regions. Seagrass meadows have traditionally been considered as biodiversity hotspots^80^. However, researchers worldwide have reported a broad range of ecological responses of benthic fauna to seagrass canopy with a number of studies that do not support the paradigm of increased diversity and abundance in seagrass beds compared to unvegetated sediments^81–85^. In the present study, depending on the variable analyzed we found evidence of the positive role of vegetation on benthic communities but responses were inconsistent denoting the influence of local scale processes reflective of differences in the environmental characteristics. For example, the combination of characteristics such as the nature of the meadow (i.e., multispecific versus monospecific; species composition^28^, shoot density and biomass^86^, local hydrodynamics and environmental conditions^37^, size of the meadow^87^, location of sample collection (i.e., “edge-effect”, the increase in diversity or abundance in the borders of the meadow), environmental variables within the meadow^88,89^, seasonality^90^ and light regulation^91^, create specific environmental and biological conditions at the local scale that might change the direction and magnitude of the observed responses. The variability in the patterns across the three sites does not allow us to disentangle the relative contribution of each factor in explaining the benthic patterns. For example, in location C, higher similarities were noted among different treatments (e.g., unvegetated versus vegetated), than between the same treatments in different locations (e.g., unvegetated location A versus unvegetated location C). This was particularly evident for benthic bacterial communities and has been previously reported for invertebrates^92^.

### Spatial patterns of diversity

Our results provide mixed evidence of the positive effect of seagrass cover on macrobenthic faunal density, as a primary influence of local environmental conditions over cover was apparent. Nevertheless, our ordination analysis showed that both macrofauna and bacteria communities in unvegetated sediments tended to cluster differently from those with the presence of seagrasses. Indeed, there was some evidence to support the theory of higher diversity in seagrass beds, with a higher number of exclusive macroinvertebrate taxa being recorded in the highest density seagrass treatment (but not benthic bacteria that peaked at mixed meadows). There are several plausible reasons that can contribute to the high variability in biological patterns. One of them is the temporal dynamics of the seagrass canopy. As demonstrated for temperate regions, significant positive effects of vegetation on diversity and biomass of macrobenthic communities may only be detected when seagrass cover reaches its maximum^90^. Therefore, a sound understanding of the seasonal dynamics of seagrass meadows is critical for a thorough assessment of the effect of seagrass cover in shaping benthic communities. Even at low latitude regions, seasonal differences in seagrass growth are noticeable^93^. Regarding benthic bacteria, biodiversity assessed by means of the number of exclusive taxa does not seem to be promoted by the presence of seagrasses as both unvegetated and mixed meadows harbored higher number of species compared to seagrass treatments.

A second potential explanation is the focus of the present study on sediment samples and the associated communities rather than those more dependent on above sediment niches such as leaves. The method used for collecting the samples (hand corer) is more appropriate for infaunal organisms. Macroinvertebrates living in sediments have been suggested to be less affected by fragmentation than large motile fauna, which usually display epibenthic behavior and are more associated with the aboveground biomass. Frost et al.^27^ suggested that infaunal species are less likely to show species-specific responses resulting in small shifts in community composition rather than clear changes in density as found for epifauna. Even though differences between unvegetated and vegetated treatments were less evident for bacteria, in one of the locations, significant changes were still observed between the two most contrasting treatments (unvegetated and SHD). One of the reasons may be related to the responses of bacteria to different below ground niches associated with the seagrasses^94^. The strategy used in the present study to establish the different treatments was based on the above ground density but a posteriori we realized that below ground biomass of the root system was not always consistent with the pre-established treatment. This might have resulted in high variability in the communities and the lack of significant changes related to treatment. However, Holmer et al.^95^ showed that the stable isotope signals in bacteria inhabiting unvegetated patches was similar to those of adjacent seagrass meadows, indicating that seagrass detritus and exudates may still contribute substantially to available organic matter even in distant, unvegetated patches.

The present results suggest that DOM supplied by seagrass meadows to the water column may fuel the pelagic microbial communities of unvegetated areas nearby (DOM values were consistently above 100 µmol C L^−1^ and higher than in shallow nearby waters influenced by urban settlement^62^), ultimately attenuating the difference in abundance of free-living planktonic bacteria between treatments. Organic matter detritus from the vegetated meadows may also become a source of carbon for sediment bacteria inhabiting unvegetated patches nearby, as shown in Holmer et al.^95^. Yet, this was not quantified in the present study. Humic DOM components also showed a positive and significant relationship with Chl *a* values but not with the protein-like components, suggesting that the humic material from freshly produced DOM accumulated while the protein material was being rapidly consumed by heterotrophic prokaryotes. Heterotrophic bacterioplankton was, indeed, one order of magnitude more abundant than their autotrophic counterparts. Although mean values only reached 3 × 10^5^ cells mL^−1^ in the SLD treatment (minimum values), these low surface abundances of heterotrophic bacteria seem the norm in pelagic environments from the central Red Sea, whether shallow^62^ or open waters (unpublished data). This emphasizes the need to complement the structural aspects of the community reported here with functional analysis in the future.

### Biological responses to environmental drivers

The overall changes in community composition between treatments of varying cover was attributed to the individual habitat preferences of selected species based on regression analysis. Overall, seagrass biomass had a positive effect on the abundance of dominant benthic taxa while fine particles (i.e., > 63 μm fraction) and metals had a primarily negative effect. Previously, researchers have found a positive effect of fragmentation on the density and species richness when compared to homogenous bottoms^96^. Dense seagrass meadows do not necessarily support high benthic diversity and abundance. The broad range of ecological traits observed in macroinvertebrates may contribute to the inconsistency in patterns that have been reported in the literature regarding the relationship between canopy and associated communities. For example, large tube-dwelling and burrowing species can be affected by high shoot density (and root-rhizome mat) that will limit their capacity to penetrate into the sediment^97^. However, complex and well-developed rhizome structures may not affect small polychaetes, also tube-dwellers, which do not require big areas in the sediment to build their tubes^19^. Seagrass bed cover can also limit the movement of mobile organisms^82^ and ultimately affect their ability to escape from predators. In the present study, shoot biomass was most often associated with increases in the abundance of some macrobenthic taxa, whose traits can be favored by the presence of the seagrasses. Based on the regression analysis, we observed that certain taxa were preferentially associated with seagrass habitats. Taxa that were positively associated with higher seagrass biomass included polychaetes from the families Eunicidae and Syllidae (mostly mobile and carnivores) as well as Terebellidae and Capitellidae (sessile or of limited mobility, mostly suspension feeders/detritivores). The increase in the trophic trait suspension feeders/detritivores in areas of higher seagrass was not surprising as flow rates within seagrass meadows have been demonstrated to be reduced resulting in higher rates of deposition of organic matter providing food source for these organisms^98–100^. The analysis of the exclusive species associated with each treatment also showed a prevalence of gammarid amphipods associated with high density of seagrasses. These are preferential prey items for fish^101,102^ and higher abundance of these crustaceans has previously been reported for seagrass beds when compared to unvegetated sediments possibly due to the protection that seagrass beds provide^83,103^. Interestingly, the bacterial genera *Tistlia* and *Pelobacter*, both known for nitrogen fixation^104–106^, were also associated with higher seagrass cover. Both genera may form a symbiotic relationship with the seagrass rhizome by providing a supply of fixed nitrogen to the growing seagrass and this relationship has previously been shown for *Pelobacter*^105^. In contrast, the bivalve *Cardiolucina semperiana* was negatively associated with both seagrass biomass and TOC possibly due to seagrass roots limiting its population density.

## Conclusion

The present findings emphasize the spatially dynamic nature and complex interplay of ecological and environmental processes driving benthic communities in a tropical coastal lagoon with heterogeneous bottoms. It is known that habitat loss or fragmentation can affect the resilience and persistence of vegetated habitats^107^ and in turn influence faunal connectivity among habitats, species diversity and ecological interactions^108,109^. Variables found to be important in predicting the response of benthic communities were related to processes working across multiple spatial scales.

Conservation strategies should prioritize connectivity between habitats to maintain natural migration of species^110^ and to preserve species that are restricted to mature habitats^111 and references therein^. Conservation measures should thus consider both vegetated and unvegetated habitats, which will also allow for the evaluation of potential changes associated with anthropogenic and natural impacts given the dynamic nature of the ecological linkages across the seascape^112,113^. Considering that only approximately 25% of invertebrate taxa were shared among treatments or locations, with 11% shared OTUs for benthic bacteria sampled, and that high density treatments supported the highest number of exclusive taxa for macroinvertebrates but not bacteria (highest at unvegetated and mixed meadows), sustaining ecological connectivity across the seascape is therefore critical in maintaining the biodiversity at regional scales.

## Supplementary information

Supplementary Figure S1

Supplementary Table S1

Supplementary Table S2

## References

[1] Birkeland, C. & Grosenbaugh, D. Ecological interactions between tropical coastal ecosystems. in *UNEP Regional Seas Reports and Studies,* Vol. 73 (PNUMA, 1985).

[2] Moberg F, Rönnbäck P (2003). Ecosystem services of the tropical seascape: interactions, substitutions and restoration. Ocean Coast. Manag..

[3] Gladstone W, Nagelkerken I (2009). Conservation and management of tropical coastal ecosystems. Ecological Connectivity Among Tropical Coastal Ecosystems.

[4] Berkström C (2012). Exploring ‘knowns’ and ‘unknowns’ in tropical seascape connectivity with insights from East African coral reefs. Estuar. Coast. Shelf Sci..

[5] Ogden, J. C. The influence of adjacent systems on the structure and function of coral reefs. In *Proceedings of the 6th International Coral Reef Symposium*, Vol. 1, 123–129 (1988).

[6] Nagelkerken I (2000). Importance of mangroves, seagrass beds and the shallow coral reef as a nursery for important coral reef fishes, using a visual census technique. Estuar. Coast. Shelf Sci..

[7] Grober-Dunsmore R, Pittman SJ, Caldow C, Kendall MS, Frazer TK, Nagelkerken I (2009). A landscape ecology approach for the study of ecological connectivity across tropical marine seascapes. Ecological Connectivity Among Tropical Coastal Ecosystems.

[8] Hemminga MA, Duarte CM (2000). Seagrass ecology.

[9] Short F, Carruthers T, Dennison W, Waycott M (2007). Global seagrass distribution and diversity: a bioregional model. J. Exp. Mar. Biol. Ecol..

[10] Knowles LL, Bell SS (1998). The influence of habitat structure in faunal-habitat associations in a Tampa Bay seagrass system Florida. Bull. Mar. Sci..

[11] Connolly RM, Hindell JS (2006). Review of nekton patterns and ecological processes in seagrass landscapes. Estuar. Coast. Shelf Sci..

[12] Horinouchi M (2007). Review of the effects of within-patch scale structural complexity on seagrass fishes. J. Exp. Mar. Biol. Ecol..

[13] Gacia E, Duarte CM, Middelburg JJ (2002). Carbon and nutrient deposition in a Mediterranean seagrass (*Posidonia oceanica*) meadow. Limnol. Oceanogr..

[14] Hendriks IE, Sintes T, Bouma TJ, Duarte CM (2008). Experimental assessment and modeling evaluation of the effects of the seagrass *Posidonia oceanica* on flow and particle trapping. Mar. Ecol. Prog. Ser..

[15] Beck MW (2001). The identification, conservation, and management of estuarine and marine nurseries for fish and invertebrates: a better understanding of the habitats that serve as nurseries for marine species and the factors that create site-specific variability in nursery quality will improve conservation and management of these areas. AIBS Bull..

[16] Heck KL, Hays G, Orth RJ (2003). Critical evaluation of the nursery role hypothesis for seagrass meadows. Mar. Ecol. Prog. Ser..

[17] Boström C, Jackson EL, Simenstad CA (2006). Seagrass landscapes and their effects on associated fauna: a review. Estuar. Coast. Shelf Sci..

[18] Unsworth RKF, Cullen LC (2010). Recognising the necessity for Indo-Pacific seagrass conservation. Conserv. Lett..

[19] Leopardas V, Uy W, Nakaoka M (2014). Benthic macrofaunal assemblages in multispecific seagrass meadows of the southern Philippines: variation among vegetation dominated by different seagrass species. J. Exp. Mar. Biol. Ecol..

[20] Waycott M (2009). Accelerating loss of seagrasses across the globe threatens coastal ecosystems. PNAS.

[21] Short FT, Wyllie-Echeverria S (1996). Natural and human-induced disturbance of seagrasses. Environ. Conserv..

[22] Short FT (2011). Extinction risk assessment of the world’s seagrass species. Biol. Conserv..

[23] Duarte CM (2002). The future of seagrass meadows. Environ. Conserv..

[24] Hastings K, Hesp P, Kendrick GA (1995). Seagrass loss associated with boat moorings at Rottnest Island, Western Australia. Ocean Coast. Manag..

[25] Orth RJ, Luckenbach ML, Marion SR, Moore KA, Wilcox DJ (2006). Seagrass recovery in the Delmarva Coastal Bays, USA. Aquat. Bot..

[26] Ruiz JM, Romero J (2001). Effects of in situ experimental shading on the Mediterranean seagrass *Posidonia oceanica*. Mar. Ecol. Prog. Ser..

[27] Frost MT, Rowden AA, Attrill MJ (1999). Effect of habitat fragmentation on the macroinvertebrate infaunal communities associated with the seagrass *Zostera marina* L. Aquat. Conserv. Mar. Freshw. Ecosyst..

[28] Hovel KA (2003). Habitat fragmentation in marine landscapes: relative effects of habitat cover and configuration on juvenile crab survival in California and North Carolina seagrass beds. Biol. Conserv..

[29] Hovel KA, Lipcius RN (2002). Effects of seagrass habitat fragmentation on juvenile blue crab survival and abundance. J. Exp. Mar. Biol. Ecol..

[30] Thomas CD, Mallorie HC (1985). Rarity, species richness and conservation: butterflies of the Atlas Mountains in Morocco. Biol. Conserv..

[31] Fahrig L (2003). Effects of habitat fragmentation on biodiversity. Ann. Rev. Ecol. Evol. Syst..

[32] Fischer J, Lindenmayer DB (2007). Landscape modification and habitat fragmentation: a synthesis. Glob. Ecol. Biogeogr..

[33] Link J (2002). Does food web theory work for marine ecosystems?. Mar. Ecol. Prog. Ser..

[34] Peterson BJ, Thompson KR, Cowan JH, Heck KL (2001). Comparison of predation pressure in temperate and subtropical seagrass habitats based on chronographic tethering. Mar. Ecol. Prog. Ser..

[35] Sweatman JL, Layman CA, Fourqurean JW (2017). Habitat fragmentation has some impacts on aspects of ecosystem functioning in a sub-tropical seagrass bed. Mar. Environ. Res..

[36] Williams JA (2016). Seagrass fragmentation impacts recruitment dynamics of estuarine-dependent fish. J. Exp. Mar. Biol. Ecol..

[37] Bell SS, Brooks RA, Robbins BD, Fonseca MS, Hall MO (2001). Faunal response to fragmentation in seagrass habitats: implications for seagrass conservation. Biol. Conserv..

[38] McCloskey RM, Unsworth RKF (2015). Decreasing seagrass density negatively influences associated fauna. PeerJ.

[39] Ubertini M (2012). Spatial variability of benthic-pelagic coupling in an estuary ecosystem: consequences for microphytobenthos resuspension phenomenon. PLoS ONE.

[40] Welsh DT (2000). Nitrogen fixation in seagrass meadows: regulation, plant–bacteria interactions and significance to primary productivity. Ecol. Lett..

[41] Alongi DM (1994). The role of bacteria in nutrient recycling in tropical mangrove and other coastal benthic ecosystems. Hydrobiologia.

[42] Harrison PG (1989). Detrital processing in seagrass systems: a review of factors affecting decay rates, remineralization and detritivory. Aquat. Bot..

[43] Mateo MA, Romero J (1997). Detritus dynamics in the seagrass *Posidonia oceanica*: elements for an ecosystem carbon and nutrient budget. Mar. Ecol. Prog. Ser..

[44] Barrón C, Apostolaki ET, Duarte CM (2014). Dissolved organic carbon fluxes by seagrass meadows and macroalgal beds. Front. Mar. Sci..

[45] Wetzel RG, Penhale PA (1979). Transport of carbon and excretion of dissolved organic carbon by leaves and roots/rhizomes in seagrasses and their epiphytes. Aquat. Bot..

[46] Martin BC (2018). Low light availability alters root exudation and reduces putative beneficial microorganisms in seagrass roots. Front. Microbiol..

[47] Danovaro R (1996). Detritus-Bacteria-Meiofauna interactions in a seagrass bed (*Posidonia oceanica*) of the NW Mediterranean. Mar. Biol..

[48] Lohrer AM, Thrush SF, Gibbs MM (2004). Bioturbators enhance ecosystem function through complex biogeochemical interactions. Nature.

[49] Rosenberg R (2001). Marine benthic faunal successional stages and related sedimentary activity. Sci. Marina.

[50] Austen MC (2002). Biodiversity links above and below the marine sediment–water interface that may influence community stability. Biodivers. Conserv..

[51] Fanjul E, Bazterrica MC, Escapa M, Grela MA, Iribarne O (2011). Impact of crab bioturbation on benthic flux and nitrogen dynamics of Southwest Atlantic intertidal marshes and mudflats. Estuar. Coast. Shelf Sci..

[52] Forster S, Graf G (1995). Impact of irrigation on oxygen flux into the sediment: intermittent pumping by *Callianassa subterranea* and “piston-pumping” by *Lanice conchilega*. Mar. Biol..

[53] Snelgrove PVR (1998). The biodiversity of macrofaunal organisms in marine sediments. Biodivers. Conserv..

[54] Hyndes GA, Lavery PS (2005). Does transported seagrass provide an important trophic link in unvegetated, nearshore areas?. Estuar. Coast. Shelf Sci..

[55] Jones DA, Ghamrawy M, Wahbeh MI, Edwards AJ, Head SM (1987). Littoral and shallow subtidal environments. Red Sea.

[56] Ruiz-Compean P (2017). Baseline evaluation of sediment contamination in the shallow coastal areas of Saudi Arabian Red Sea. Mar. Pollut. Bull..

[57] Bologna PAX, Heck KL (2002). Impact of habitat edges on density and secondary production of seagrass-associated fauna. Estuaries.

[58] Calleja ML, Al-Otaibi N, Morán XAG (2019). Dissolved organic carbon contribution to oxygen respiration in the central Red Sea. Sci. Rep..

[59] Stedmon CA, Markager S, Bro R (2003). Tracing dissolved organic matter in aquatic environments using a new approach to fluorescence spectroscopy. Mar. Chem..

[60] Coble PG (2007). Marine optical biogeochemistry: the chemistry of ocean color. Chem. Rev..

[61] Gasol JM, Morán XAG, McGenity TJ, Timmis KN, Nogales B (2015). Flow cytometric determination of microbial abundances and its use to obtain indices of community structure and relative activity. Hydrocarbon and Lipid Microbiology Protocols: Single-Cell and Single-Molecule Methods.

[62] Silva L (2019). Low abundances but high growth rates of coastal heterotrophic bacteria in the Red Sea. Front. Microbiol..

[63] Leray M, Knowlton N (2015). DNA barcoding and metabarcoding of standardized samples reveal patterns of marine benthic diversity. PNAS.

[64] Klindworth A (2013). Evaluation of general 16S ribosomal RNA gene PCR primers for classical and next-generation sequencing-based diversity studies. Nucl. Acids Res..

[65] Oksanen, J. *et al.* Vegan: community ecology package. R package version 2.5-2, (2018).

[66] R Core Team. *R: A Language and Environment for Statistical Computing* (R Foundation for Statistical Computing, Vienna, 2018).

[67] Lê S, Josse J, Husson F (2008). FactoMineR: a package for multivariate analysis. J. Stat. Softw..

[68] Goslee SC, Urban DL (2007). The ecodist package for dissimilarity-based analysis of ecological data. J. Stat. Softw..

[69] Chen, H. *VennDiagram: Generate High-Resolution Venn and Euler Plots* (2018).

[70] Clarke, K. R. & Gorley, R. N. PRIMER v7: User Manual/Tutorial, PRIMER-E: Plymouth (2015).

[71] Callahan BJ (2016). DADA2: high-resolution sample inference from Illumina amplicon data. Nat. Methods.

[72] Pruesse E (2007). SILVA: a comprehensive online resource for quality checked and aligned ribosomal RNA sequence data compatible with ARB. Nucl. Acids Res..

[73] McMurdie PJ, Holmes S (2013). phyloseq: an R package for reproducible interactive analysis and graphics of microbiome census data. PLoS ONE.

[74] Keeley N, Wood SA, Pochon X (2018). Development and preliminary validation of a multi-trophic metabarcoding biotic index for monitoring benthic organic enrichment. Ecol. Indic..

[75] Lobelle D, Kenyon EJ, Cook KJ, Bull JC (2013). Local competition and metapopulation processes drive long-term seagrass-epiphyte population dynamics. PLoS ONE.

[76] Ávila E, Yáñez B, Vazquez-Maldonado LE (2015). Influence of habitat structure and environmental regime on spatial distribution patterns of macroinvertebrate assemblages associated with seagrass beds in a southern Gulf of Mexico coastal lagoon. Mar. Biol. Res..

[77] Barnes RSK, Hendy IW (2015). Seagrass-associated macrobenthic functional diversity and functional structure along an estuarine gradient. Estuar. Coast. Shelf Sci..

[78] York PH, Hyndes GA, Bishop MJ, Barnes RSK, Larkum AWD, Kendrick GA, Ralph PJ (2018). Faunal assemblages of seagrass ecosystems. Seagrasses of Australia: Structure, Ecology and Conservation.

[79] Magni P, Como S, Kamijo A, Montani S (2017). Effects of *Zostera marina* on the patterns of spatial distribution of sediments and macrozoobenthos in the boreal lagoon of Furen (Hokkaido, Japan). Mar. Environ. Res..

[80] Thomsen MS (2018). Secondary foundation species enhance biodiversity. Nat. Ecol. Evol..

[81] Attrill MJ, Strong JA, Rowden AA (2000). Are macroinvertebrate communities influenced by seagrass structural complexity?. Ecography.

[82] Lee SY, Fong CW, Wu RSS (2001). The effects of seagrass (*Zostera japonica*) canopy structure on associated fauna: a study using artificial seagrass units and sampling of natural beds. J. Exp. Mar. Biol. Ecol..

[83] Nakamura Y, Sano M (2005). Comparison of invertebrate abundance in a seagrass bed and adjacent coral and sand areas at Amitori Bay, Iriomote Island, Japan. Fish. Sci..

[84] Barrio Froján CRS (2009). The importance of bare marine sedimentary habitats for maintaining high polychaete diversity and the implications for the design of marine protected areas. Aquat. Conserv. Mar. Freshw. Ecosyst..

[85] Barnes RSK, Barnes MKS (2014). Spatial uniformity of biodiversity is inevitable if the available species are distributed independently of each other. Mar. Ecol. Prog. Ser..

[86] Webster PJ, Rowden AA, Attrill MJ (1998). Effect of shoot density on the infaunal macro-invertebrate community within a *Zostera marina* seagrass bed. Estuar. Coast. Shelf Sci..

[87] Bowden DA, Rowden AA, Attrill MJ (2001). Effect of patch size and in-patch location on the infaunal macroinvertebrate assemblages of *Zostera marina* seagrass beds. J. Exp. Mar. Biol. Ecol..

[88] Turner SJ (1999). Seagrass patches and landscapes: the influence of wind-wave dynamics and hierarchical arrangements of spatial structure on macrofaunal seagrass communities. Estuaries.

[89] Tanner JE (2005). Edge effects on fauna in fragmented seagrass meadows. Austral Ecol..

[90] Włodarska-Kowalczuk M, Jankowska E, Kotwicki L, Balazy P (2014). Evidence of season-dependency in vegetation effects on macrofauna in temperate seagrass meadows (Baltic Sea). PLoS ONE.

[91] Calleja ML, Barrón C, Hale JA, Frazer TK, Duarte CM (2006). Light regulation of benthic sulfate reduction rates mediated by seagrass (*Thalassia testudinum*) metabolism. Estuar. Coasts J ERF.

[92] Barnes RSK, Barnes MKS (2012). Shore height and differentials between macrobenthic assemblages in vegetated and unvegetated areas of an intertidal sandflat. Estuar. Coast. Shelf Sci..

[93] Agawin NSR, Duarte CM, Fortes MD, Uri JS, Vermaat JE (2001). Temporal changes in the abundance, leaf growth and photosynthesis of three co-occurring Philippine seagrasses. J. Exp. Mar. Biol. Ecol..

[94] Pereg LL, Lipkin Y, Sar N (1994). Different niches of the *Halophila stipulacea* seagrass bed harbor distinct populations of nitrogen fixing bacteria. Mar. Biol..

[95] Holmer M, Duarte C, Boschker H, Barrón C (2004). Carbon cycling and bacterial carbon sources in pristine and impacted Mediterranean seagrass sediments. Aquat. Microb. Ecol..

[96] Barberá-Cebrián C, Sánchez-Jerez P, Ramos-Esplá A (2002). Fragmented seagrass habitats on the Mediterranean coast, and distribution and abundance of mysid assemblages. Mar. Biol..

[97] Ringold P (1979). Burrowing, root mat density, and the distribution of fiddler crabs in the eastern United States. J. Exp. Mar. Biol. Ecol..

[98] Ricart AM (2015). Variability of sedimentary organic carbon in patchy seagrass landscapes. Mar. Pollut. Bull..

[99] Samper-Villarreal J, Lovelock CE, Saunders MI, Roelfsema C, Mumby PJ (2016). Organic carbon in seagrass sediments is influenced by seagrass canopy complexity, turbidity, wave height, and water depth. Limnol. Oceanogr..

[100] Serra T, Oldham C, Colomer J (2018). Local hydrodynamics at edges of marine canopies under oscillatory flows. PLoS ONE.

[101] Choat JH, Kingett PD (1982). The influence of fish predation on the abundance cycles of an algal turf invertebrate fauna. Oecologia.

[102] Nakamura Y, Horinouchi M, Nakai T, Sano M (2003). Food habits of fishes in a seagrass bed on a fringing coral reef at Iriomote Island, southern Japan. Ichthyol. Res..

[103] Eklöf JS, de la Torre Castro M, Adelsköld L, Jiddawi NS, Kautsky N (2005). Differences in macrofaunal and seagrass assemblages in seagrass beds with and without seaweed farms. Estuar. Coast. Shelf Sci..

[104] Díaz-Cárdenas C, Patel BKC, Baena S (2010). Tistlia consotensisgen. nov., sp. an aerobic, chemoheterotrophic, free-living, nitrogen-fixing alphaproteobacterium, isolated from a Colombian saline spring. Int. J. Syst. Evol. Microbiol..

[105] Sun F, Zhang X, Zhang Q, Liu F, Zhang J, Gong J (2015). Seagrass (*Zostera marina*) colonization promotes the accumulation of diazotrophic bacteria and alters the relative abundances of specific bacterial lineages involved in benthic carbon and sulfur cycling. Appl. Environ. Microbiol..

[106] Brown SM, Jenkins BD (2014). Profiling gene expression to distinguish the likely active diazotrophs from a sea of genetic potential in marine sediments. Environ. Microbiol..

[107] Santos R, Lirman D, Pittman S (2015). Long-term spatial dynamics in vegetated seascapes: fragmentation and habitat loss in a human-impacted subtropical lagoon. Mar. Ecol..

[108] Irlandi E, Crawford M (1997). Habitats linkages: the effect of intertidal saltmarshes and adjacent habitats on abundance, movement and growth of an estuarine fish. Oecologia.

[109] Boström C, Pittman SJ, Simenstad C, Kneib RT (2011). Seascape ecology of coastal biogenic habitats: advances, gaps, and challenges. Mar. Ecol. Prog. Ser..

[110] Mumby PJ (2006). Connectivity of reef fish between mangroves and coral reefs: algorithms for the design of marine reserves at seascape scales. Biol. Conserv..

[111] Haila Y (2002). A conceptual genealogy of fragmentation research: from island biogeography to landscape ecology. Ecol. Appl..

[112] Barnes RSK (2013). Distribution patterns of macrobenthic biodiversity in the intertidal seagrass beds of an estuarine system, and their conservation significance. Biodivers. Conserv..

[113] Barnes RSK, Hamylton S (2016). On the very edge: faunal and functional responses to the interface between benthic seagrass and unvegetated sand assemblages. Mar. Ecol. Prog. Ser..

