# Supplementary material for: The role of seagrass vegetation and local environmental conditions in shaping benthic bacterial and macroinvertebrate communities in a tropical coastal lagoon
Source: Sci Rep. 2020 Aug 11;10:13550. doi: 10.1038/s41598-020-70318-1 (PMC7419567; doi:10.1038/s41598-020-70318-1)

**The role of seagrass vegetation and local environmental conditions in shaping benthic bacterial and macroinvertebrate communities in a tropical coastal lagoon**

## Alsaffar Z.^1,2^, Pearman J.K.^1,3^, Cúrdia J.^1^, Ellis J.^1,4^, Calleja M.Ll.^1,5^, Ruiz-Compean P.^1^, Roth F.^1,6,7^, Villalobos R.^1^, Jones B.H.^1^, Morán X. A. G.^1^, Carvalho S.^1*^

^1^ King Abdullah University of Science and Technology (KAUST), Red Sea Research Center (RSRC), Biological and Environmental Sciences and Engineering (BESE), Thuwal, Saudi Arabia

^2^ King Saud University, Riyadh, Saudi Arabia

^3^ Coastal and Freshwater Group, Cawthron Institute, Nelson, New Zealand

^4^ School of Science, University of Waikato, Tauranga, New Zealand

^5^ Department of Climate Geochemistry, Max Planck Institute for Chemistry (MPIC), Mainz, Germany

^6^ Baltic Sea Centre, Stockholm University, Stockholm, Sweden

^7^ Faculty of Biological and Environmental Sciences, Tvärminne Zoological Station, University of Helsinki, Helsinki, Finland

S1. Schematic representation of collinearity between measured environmental variables representing grain size, trace elements, dissolved inorganic nutrients, productivity proxies and other nutrients. Correlation (Pearson’s r) values presented for correlated variables. Green color denotes positive correlations and red negative. The variables in green boxes were used in the Principal Component Analysis (PCA) to reduce multicollinearity.


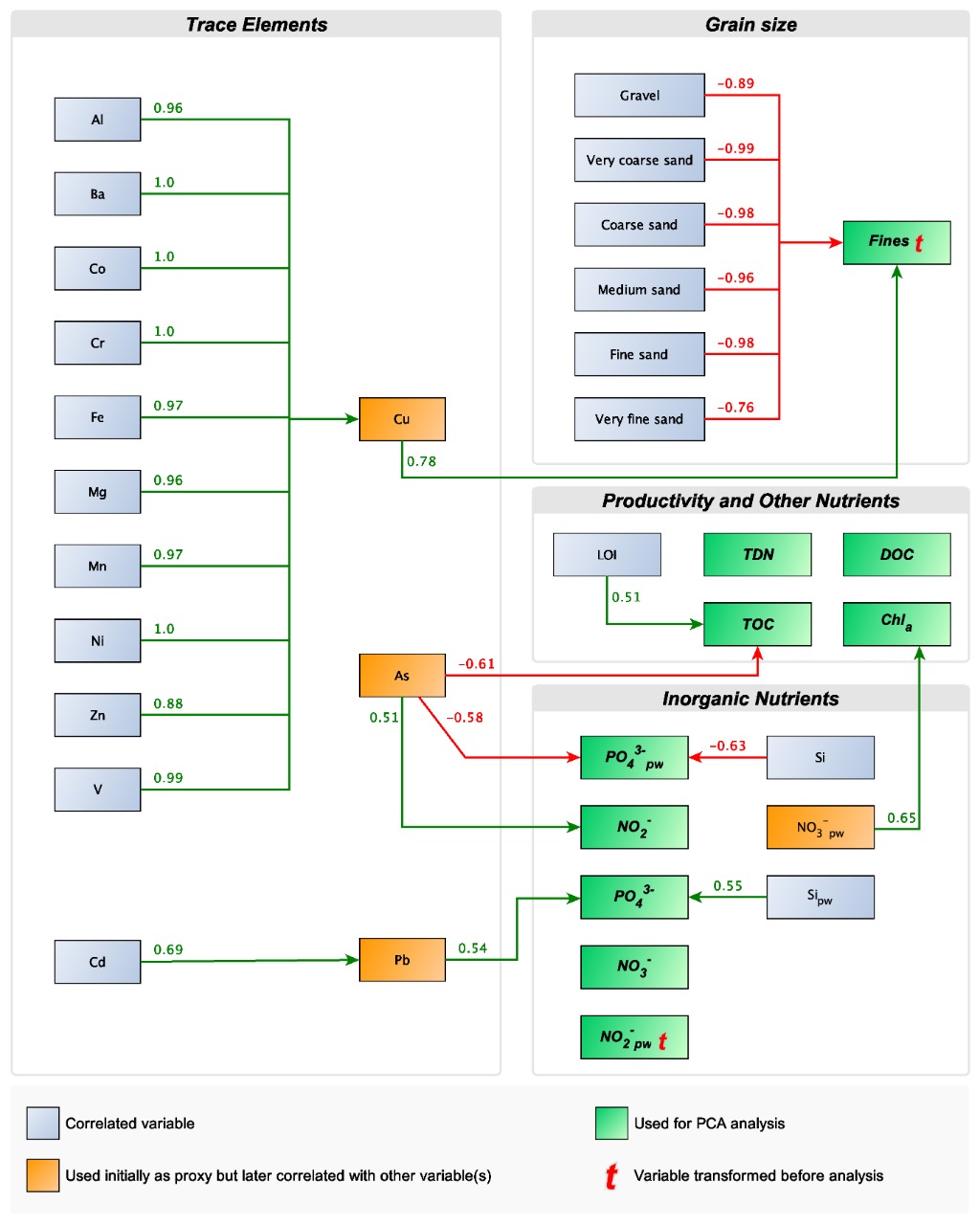

Supplement: Supplementary file 1 — Supplementary Figure S1 [file 41598_2020_70318_MOESM1_ESM.docx]
