# Supplementary material for: The role of seagrass vegetation and local environmental conditions in shaping benthic bacterial and macroinvertebrate communities in a tropical coastal lagoon
Source: Sci Rep. 2020 Aug 11;10:13550. doi: 10.1038/s41598-020-70318-1 (PMC7419567; doi:10.1038/s41598-020-70318-1)
Supplement: Supplementary file 2 — Supplementary Table S1 [file 41598_2020_70318_MOESM2_ESM.docx]

**The role of seagrass vegetation and local environmental conditions in shaping benthic bacterial and macroinvertebrate communities in a tropical coastal lagoon**

## Alsaffar Z.^1,2^, Pearman J.K.^1,3^, Cúrdia J.^1^, Ellis J.^1,4^, Calleja M.Ll.^1,5^, Ruiz-Compean P.^1^, Roth F.^1,6,7^, Villalobos R.^1^, Jones B.H.^1^, Morán X. A. G.^1^, Carvalho S.^1*^

^1^ King Abdullah University of Science and Technology (KAUST), Red Sea Research Center (RSRC), Biological and Environmental Sciences and Engineering (BESE), Thuwal, Saudi Arabia

^2^ King Saud University, Riyadh, Saudi Arabia

^3^ Coastal and Freshwater Group, Cawthron Institute, Nelson, New Zealand

^4^ School of Science, University of Waikato, Tauranga, New Zealand

^5^ Department of Climate Geochemistry, Max Planck Institute for Chemistry (MPIC), Mainz, Germany

^6^ Baltic Sea Centre, Stockholm University, Stockholm, Sweden

^7^ Faculty of Biological and Environmental Sciences, Tvärminne Zoological Station, University of Helsinki, Helsinki, Finland

Table S1. Two-way ANOVA results and pair-wise tests for abundance and number of taxa (macrofauna) and number of operational taxonomic units (OTUs) for bacteria. Unvegetated (UnV); seagrass and algae (SA) mixed meadows; seagrass low density (SLD); seagrass high density (SHD). A, B and C denote locations. Significant values are shown in bold.

|  | **Macrofauna** | | | | | | | |  | **Bacteria** | | | |
| --- | --- | --- | --- | --- | --- | --- | --- | --- | --- | --- | --- | --- | --- |
|  |  | **Abundance** | | |  | **Number of taxa (logeS)** | | |  | **Number of OTUs** | | | |
| **Source** | **df** | **MS** | **F** | **P** |  | **MS** | **F** | **P** |  | df | MS | F | P |
| **Treatment (Tr)** | 3 | 0.5366 | 1.558 | 0.225 |  | 0.1458 | 0.934 | 0.440 |  | 3 | 0.1458 | 2.553 | 0.076 |
| **Location (Lo)** | 2 | 2.4808 | **7.201** | **0.004** |  | 2.2631 | **14.491** | **<0.001** |  | 2 | **2.2631** | **18.7911** | **<0.001** |
| **Tr x Lo** | 6 | 0.2943 | 0.854 | 0.541 |  | 0.1584 | 1.015 | 0.439 |  | 6 | **0.1584** | **2.5462** | **0.043** |
| **Res** | 24 | 0.3445 |  |  |  | 0.1562 |  |  |  | 28 |  |  |  |
|  |  |  |  |  |  |  |  |  |  |  |  |  |  |
|  |  |  |  |  |  |  |  |  |  | *Term 'Tr x Lo'* | | | |
|  | |  |  |  |  |  |  |  |  | Within level 'Unv' of factor 'Treatment' | | | |
|  |  |  |  |  |  |  |  |  |  |  | Groups | t | p |
|  |  |  |  |  |  |  |  |  |  |  | A,B | 1.585 | 0.2689 |
|  |  |  |  |  |  |  | | |  |  | **A,C** | **4.516** | **0.0003** |
| *Term "Lo"* |  |  |  |  |  |  |  |  |  |  | **B,C** | **5.983** | **<0.0001** |
| Groups |  | t | p |  |  |  | t | p |  |  |  |  |  |
| A,B |  | 1.121 | 0.5107 |  |  |  | 2.555 | 0.0443 |  | Within level 'SA' of factor 'Treatment' | | | |
| **A,C** |  | **2.579** | **0.042** |  |  |  | 2.826 | 0.0244 |  |  | Groups | t | p |
| **B,C** |  | **3.701** | **0.0031** |  |  |  | **5.381** | **<0.0001** |  |  | A,B | 0.514 | 0.8650 |
|  |  |  |  |  |  |  |  |  |  |  | **A,C** | **3.523** | **0.0041** |
|  |  |  |  |  |  |  |  |  |  |  | **B,C** | **3.197** | **0.0093** |
|  |  |  |  |  |  |  |  |  |  |  |  |  |  |
|  |  |  |  |  |  |  |  |  |  | Within level 'SLD' of factor 'Treatment' | | | |
|  |  |  |  |  |  |  |  |  |  |  | Groups | t | p |
|  |  |  |  |  |  |  |  |  |  |  | A,B | 0.514 | 0.8652 |
|  |  |  |  |  |  |  |  |  |  |  | **A,C** | **3.836** | **0.0018** |
|  |  |  |  |  |  |  |  |  |  |  | **B,C** | **3.322** | **0.0068** |
|  |  |  |  |  |  |  |  |  |  |  |  |  |  |
|  |  |  |  |  |  |  |  |  |  | Within level 'SHD' of factor 'Treatment' | | | |
|  |  |  |  |  |  |  |  |  |  |  | Groups | t | p |
|  |  |  |  |  |  |  |  |  |  |  | A,B | 1.615 | 0.2561 |
|  |  |  |  |  |  |  |  |  |  |  | A,C | 0.629 | 0.8053 |
|  |  |  |  |  |  |  |  |  |  |  | B,C | 2.424 | 0.0557 |
|  |  |  |  |  |  |  |  |  |  |  |  |  |  |
|  |  |  |  |  |  |  |  |  |  | Within level 'A' of factor 'Location' | | | |
|  |  |  |  |  |  |  |  |  |  |  | Groups | t | p |
|  |  |  |  |  |  |  |  |  |  |  | UnV,SA | 2.091 | 0.1807 |
|  |  |  |  |  |  |  |  |  |  |  | UnV,SLD | 1.047 | 0.7238 |
|  |  |  |  |  |  |  |  |  |  |  | UnV,SHD | 0.555 | 0.9444 |
|  |  |  |  |  |  |  |  |  |  |  | SA,SLD | 0.889 | 0.8105 |
|  |  |  |  |  |  |  |  |  |  |  | SA.SHD | 2.491 | 0.0833 |
|  |  |  |  |  |  |  |  |  |  |  | SLD,SHD | 1.498 | 0.4518 |
|  |  |  |  |  |  |  |  |  |  | Within level 'B' of factor 'Location' | | | |
|  |  |  |  |  |  |  |  |  |  |  | Groups | t | p |
|  |  |  |  |  |  |  |  |  |  |  | UnV,SA | 0.008 | 1.0000 |
|  |  |  |  |  |  |  |  |  |  |  | UnV,SLD | 0.970 | 0.7675 |
|  |  |  |  |  |  |  |  |  |  |  | UnV,SHD | 0.440 | 0.9710 |
|  |  |  |  |  |  |  |  |  |  |  | SA,SLD | 0.962 | 0.7717 |
|  |  |  |  |  |  |  |  |  |  |  | SA,SHD | 0.432 | 0.9725 |
|  |  |  |  |  |  |  |  |  |  |  | SLD,SHD | 0.563 | 0.9423 |
|  |  |  |  |  |  |  |  |  |  | Within level 'C' of factor 'Location' | | | |
|  |  |  |  |  |  |  |  |  |  |  | Groups | t | p |
|  |  |  |  |  |  |  |  |  |  |  | UnV,SA | 0.857 | 0.8268 |
|  |  |  |  |  |  |  |  |  |  |  | UnV,SLD | 1.368 | 0.5292 |
|  |  |  |  |  |  |  |  |  |  |  | **UnV,SHD** | **3.331** | **0.0123** |
|  |  |  |  |  |  |  |  |  |  |  | SA,SLD | 0.111 | 0.9995 |
|  |  |  |  |  |  |  |  |  |  |  | SA,SHD | 1.391 | 0.5152 |
|  |  |  |  |  |  |  |  |  |  |  | SLD,SHD | 1.869 | 0.2639 |
