# Supplementary material for: The role of seagrass vegetation and local environmental conditions in shaping benthic bacterial and macroinvertebrate communities in a tropical coastal lagoon
Source: Sci Rep. 2020 Aug 11;10:13550. doi: 10.1038/s41598-020-70318-1 (PMC7419567; doi:10.1038/s41598-020-70318-1)
Supplement: Supplementary file 3 — Supplementary Table S2 [file 41598_2020_70318_MOESM3_ESM.docx]

**The role of seagrass vegetation and local environmental conditions in shaping benthic bacterial and macroinvertebrate communities in a tropical coastal lagoon**

## Alsaffar Z.^1,2^, Pearman J.K.^1,3^, Cúrdia J.^1^, Ellis J.^1,4^, Calleja M.Ll.^1,5^, Ruiz-Compean P.^1^, Roth F.^1,6,7^, Villalobos R.^1^, Jones B.H.^1^, Morán X. A. G.^1^, Carvalho S.^1*^

^1^ King Abdullah University of Science and Technology (KAUST), Red Sea Research Center (RSRC), Biological and Environmental Sciences and Engineering (BESE), Thuwal, Saudi Arabia

^2^ King Saud University, Riyadh, Saudi Arabia

^3^ Coastal and Freshwater Group, Cawthron Institute, Nelson, New Zealand

^4^ School of Science, University of Waikato, Tauranga, New Zealand

^5^ Department of Climate Geochemistry, Max Planck Institute for Chemistry (MPIC), Mainz, Germany

^6^ Baltic Sea Centre, Stockholm University, Stockholm, Sweden

^7^ Faculty of Biological and Environmental Sciences, Tvärminne Zoological Station, University of Helsinki, Helsinki, Finland

Table S2. Two-way PERMANOVA results and pair-wise tests based on Bray-Curtis dissimilarity. Unvegetated (UnV); seagrass and algae (SA) mixed meadows; seagrass low density (SLD); seagrass high density (SHD). A, B and C denote locations. Significant values are shown in bold.

|  | **Macrofauna** | | | |  | **Bacteria** | | | |
| --- | --- | --- | --- | --- | --- | --- | --- | --- | --- |
| Source | df | MS | Pseudo-F | P(perm) |  | df | MS | Pseudo-F | P(perm) |
| **Treatment (Tr)** | 3 | 7041 | 3.1521 | 0.001 |  | 3 | 0.30875 | 1.7883 | 0.001 |
| **Location (Lo)** | 2 | 5716.5 | 2.5591 | 0.001 |  | 2 | 0.40342 | 2.3366 | 0.001 |
| **Tr x Lo** | 6 | 2475 | 1.108 | 0.251 |  | 6 | 0.22202 | 1.286 | 0.018 |
| **Res** | 24 | 2233.7 |  |  |  | 28 | 0.17265 |  |  |
| **Total** | 35 |  |  |  |  | 39 |  |  |  |
|  |  |  |  |  |  |  |  |  |  |
|  | Pair-wise comparisons | |  |  |  |  |  |  |  |
|  |  | Term "Lo" |  |  |  | Term 'Tr x Lo' |  |  |  |
|  |  | Groups | t | P(MC) |  | Within level 'C' of factor 'Treatment' | | |  |
|  |  | A,B | 1.3851 | 0.041 |  |  | A,B | 0.8986 | 0.548 |
|  |  | A,C | 1.7052 | 0.001 |  |  | A,C | 1.4346 | 0.118 |
|  |  | B,C | 1.7263 | 0.001 |  |  | B,C | 1.4561 | 0.097 |
|  |  |  |  |  |  |  |  |  |  |
|  |  |  |  |  |  | Within level 'SHD' of factor 'Treatment' | | |  |
|  |  |  |  |  |  |  | Groups | t | P(MC) |
|  |  |  |  |  |  |  | A,B | 0.91632 | 0.525 |
|  |  |  |  |  |  |  | A,C | 1.5343 | 0.084 |
|  |  |  |  |  |  |  | B,C | 1.841 | 0.018 |
|  |  |  |  |  |  |  |  |  |  |
|  |  |  |  |  |  | Within level 'SLD' of factor 'Treatment' | | |  |
|  |  |  |  |  |  |  | A,B | 1.1015 | 0.339 |
|  |  |  |  |  |  |  | A,C | 1.4977 | 0.1 |
|  |  |  |  |  |  |  | B,C | 1.2941 | 0.184 |
|  |  |  |  |  |  |  |  |  |  |
|  |  |  |  |  |  | Within level 'SA' of factor 'Treatment' | | |  |
|  |  |  |  |  |  |  | A,B | 1.0252 | 0.413 |
|  |  |  |  |  |  |  | A,C | 1.1714 | 0.317 |
|  |  |  |  |  |  |  | B,C | 1.1287 | 0.347 |
|  |  |  |  |  |  |  |  |  |  |
|  |  | Term 'Tr' |  |  |  | Within level 'A' of factor 'Location' | | |  |
|  |  | Groups | t | P(MC) |  |  | UnV,SA | 1.1533 | O.258 |
|  |  | UnV,SHD | 2.3119 | 0.001 |  |  | UnV,SHD | 1.1326 | 0.31 |
|  |  | UnV,SLD | 2.2702 | 0.001 |  |  | UnV,SLD | 1.2641 | 0.166 |
|  |  | UnV,SA | 2.1936 | 0.002 |  |  | SA,SHD | 1.1545 | 0.28 |
|  |  | SHD,SLD | 1.3056 | 0.075 |  |  | SA,SLD | 0.97685 | 0.463 |
|  |  | SHD,SA | 1.2061 | 0.153 |  |  | SHD,SLD | 1.2046 | 0.242 |
|  |  | SLD,SA | 1.0845 | 0.338 |  |  |  |  |  |
|  |  |  |  |  |  | Within level 'B' of factor 'Location' | | |  |
|  |  |  |  |  |  |  | UnV,SA | 1.2488 | 0.187 |
|  |  |  |  |  |  |  | UnV,SHD | 1.2137 | 0.229 |
|  |  |  |  |  |  |  | UnV,SLD | 1.0299 | 0.381 |
|  |  |  |  |  |  |  | SA,SHD | 1.2064 | 0.205 |
|  |  |  |  |  |  |  | SA,SLD | 1.046 | 0.37 |
|  |  |  |  |  |  |  | SHD,SLD | 0.9964 | 0.476 |
|  |  |  |  |  |  |  |  |  |  |
|  |  |  |  |  |  | Within level 'C' of factor 'Location' | | |  |
|  |  |  |  |  |  |  | UnV,SA | 1.1544 | 0.33 |
|  |  |  |  |  |  |  | UnV,SHD | 1.7021 | 0.038 |
|  |  |  |  |  |  |  | UnV,SLD | 1.4749 | 0.097 |
|  |  |  |  |  |  |  | SA,SHD | 1.3558 | 0.191 |
|  |  |  |  |  |  |  | SA,SLD | 1.2061 | 0.327 |
|  |  |  |  |  |  |  | SHD,SLD | 1.4893 | 0.098 |
